# Syntaxin-1/TI-VAMP SNAREs interact with Trk receptors and are required for neurotrophin-dependent outgrowth

**DOI:** 10.18632/oncotarget.26307

**Published:** 2018-11-13

**Authors:** Giulia Fuschini, Tiziana Cotrufo, Oriol Ros, Ashraf Muhaisen, Rosa Andrés, Joan X. Comella, Eduardo Soriano

**Affiliations:** ^1^ Department of Cell Biology, Physiology and Immunology, Faculty of Biology, University of Barcelona, 08028 Barcelona, Spain; ^2^ Centro de Investigación Biomédica en Red sobre Enfermedades Neurodegenerativas (CIBERNED), ISCIII, 28031 Madrid, Spain; ^3^ Vall d'Hebron Institute of Research (VHIR), 08035 Barcelona, Spain; ^4^ Institució Catalana de Recerca i Estudis Avançats (ICREA), 08010 Barcelona, Spain

**Keywords:** SNARE proteins, Trk receptors, exocytosis, neurotrophins, neuronal outgrowth

## Abstract

SNARE proteins are essential components of the machinery that regulates vesicle trafficking and exocytosis. Their role is critical for the membrane-fusion processes that occur during neurotransmitter release. However, research in the last decade has also unraveled the relevance of these proteins in membrane expansion and cytoskeletal rearrangements during developmental processes such as neuronal migration and growth cone extension and attraction. Neurotrophins are neurotrophic factors that are required for many cellular functions throughout the brain, including neurite outgrowth and guidance, synaptic formation, and plasticity. Here we show that neurotrophin Trk receptors form a specific protein complex with the t-SNARE protein Syntaxin 1, both *in vivo* and *in vitro*. We also demonstrate that blockade of Syntaxin 1 abolishes neurotrophin-dependent growth of axons in neuronal cultures and decreases exocytotic events at the tip of axonal growth cones. 25-kDa soluble N-ethylmaleimide-sensitive factor attachment protein and Vesicle-associated membrane protein 2 do not participate in the formation of this SNARE complex, while tetanus neurotoxin-insensitive vesicle-associated membrane protein interacts with Trk receptors; knockdown of this (v) SNARE impairs Trk-dependent outgrowth. Taken together, our results support the notion that an atypical SNARE complex comprising Syntaxin 1 and tetanus neurotoxin-insensitive vesicle-associated membrane protein is required for axonal neurotrophin function.

## INTRODUCTION

The plasma-membrane target (t) SNARE Syntaxin 1 (Sytx1), the 25-kDa soluble N-ethylmaleimide-sensitive factor attachment protein (SNAP25), and the vesicle (v)-associated membrane proteins VAMP2 and TI-VAMP are present in axonal growth cones [1–6]. It has been proposed that these molecules, together with SEC1/MUNC18-like proteins, mediate axonal growth [7]. SNARE proteins are indeed crucial for membrane fusion events. In neurons, the t-SNAREs Sytx1 and SNAP25, and the v-SNARE VAMP2 (and/or TI-VAMP) form the SNARE complex that mediates the fusion of presynaptic vesicles with the plasma membrane in axons [8, 9]. Axonal growth and guidance are fundamental events required to establish the normal patterns of connections in the central and peripheral nervous system. Directional axonal growth and extension, together with cell migration, call for cytoskeletal reorganization and stabilization [10–12], but also for membrane dynamics and insertion—processes that mediate the extension of neuronal leading edges and growth cones [13]. Indeed, a VAMP1-like protein, TI-VAMP, which is insensitive to tetanus neurotoxin and several Botulinum toxins [14, 15], plays a major role in neurite outgrowth by coordinating membrane trafficking and actin remodeling [1, 3, 9, 16]. One mechanism by which TI-VAMP leads to the growth of axons, as well as their regulation, was clarified when Sytx1 was shown to be necessary for Netrin-1-mediated axonal growth and guidance and for neuronal migration through direct interaction with the Netrin-1 receptor DCC [17, 18]. This interaction promotes the exocytosis of vesicles in axonal growth cones and leading edges of migrating cells through an atypical SNARE complex that involves TI-VAMP but not SNAP25 or VAMP2. Additionally, it has been shown that Sema3A-mediated repulsion requires the interaction of VAMP2 with Neuropilin 1 (Nrp1) and Plexin A1 (PlexA1), two essential components of the Semaphorin 3A (Sema3A) receptor [19]. These findings have led to the hypothesis that axon guidance involves asymmetric membrane trafficking across the growth cone; in particular, a localized increase in Ca^2+^ in response to guidance cues evokes SNARE-dependent exocytosis to promote attractive turning [5, 20] or the clathrin-dependent endocytosis necessary for repulsion [21], depending on whether the Ca^2+^ signal is accompanied by Ca^2+^ release from internal stores [13, 22].

Nerve growth factor (NGF), brain-derived neurotrophic factor (BDNF), neurotrophin-3 (NT-3), and neurotrophin-4 (NT-4) are members of the neurotrophin family of growth factors. They are all polypeptides that support the growth, differentiation and survival of neurons in the developing nervous system and maintain neurons in the mature nervous system [23–25]. Neurotrophins bind to a receptor called p75NTR and to one of the three tropomyosin-related kinase (Trk) receptors. NGF binds to TrkA, BDNF and NT4 to TrkB, and NT3 to TrkC. It is the differential expression and distribution of these receptors and their ligands that allow neurotrophins to exert many different functions [25–27].

Indeed, neurotrophins play an important role in regulating various processes, including neural survival, development, function, and plasticity [28–30]. Many lines of evidence have also demonstrated that neurotrophic factors promote both axon and dendrite growth [31–33]. The absence of TrkB and TrkC signaling interferes with the functional maturation of the presynaptic machinery by altering the number of synaptic vesicles or their exocytotic/endocytotic cycle or both [34].

Moreover, the original discovery that Trks are oncogenes has led to the association of their expression with the biology of tumors; their expression and signaling cascades are often modified not only in tumors of neural origin but also in carcinomas, myelomas, and prostate and lymphoid tumors [35, 36].

In previous studies, we demonstrated that the t-SNARE protein Sytx1 is involved in DCC/Netrin-1-dependent chemoattraction [17, 18] and that Sytx1 and other SNARE proteins are required for the correct formation of motoneuronal tracts *in vivo* [37]. Those findings, together with those of other studies, suggest that the crosstalk between SNARE proteins and the receptors that mediate guidance and outgrowth could be a general mechanism.

SNARE protein complexes are also overexpressed in several cancer subtypes, thereby once again suggesting a strong link between cancer and neural development. In particular, HER2-enriched breast tumors overexpress Sytx1A [38]. Additionally, recent results have revealed that blocking Sytx1 function in glioblastoma cells halts their growth and that human glioblastoma cells with inhibited Sytx1 function give rise to brain tumors that are up to eight times smaller than control tumor cells [39].

Here we addressed whether Trk-mediated neurotrophin effects on neurite outgrowth require the involvement of SNARE proteins. We show that Trk receptors interact with Sytx1 and TI-VAMP and that these SNARE proteins are necessary for neurotrophins to induce neurite outgrowth via exocytosis.

## RESULTS

### Trk receptors associate with the t-SNARE Sytx1A/1B *in vitro* and *in vivo*

**Figure 1:**
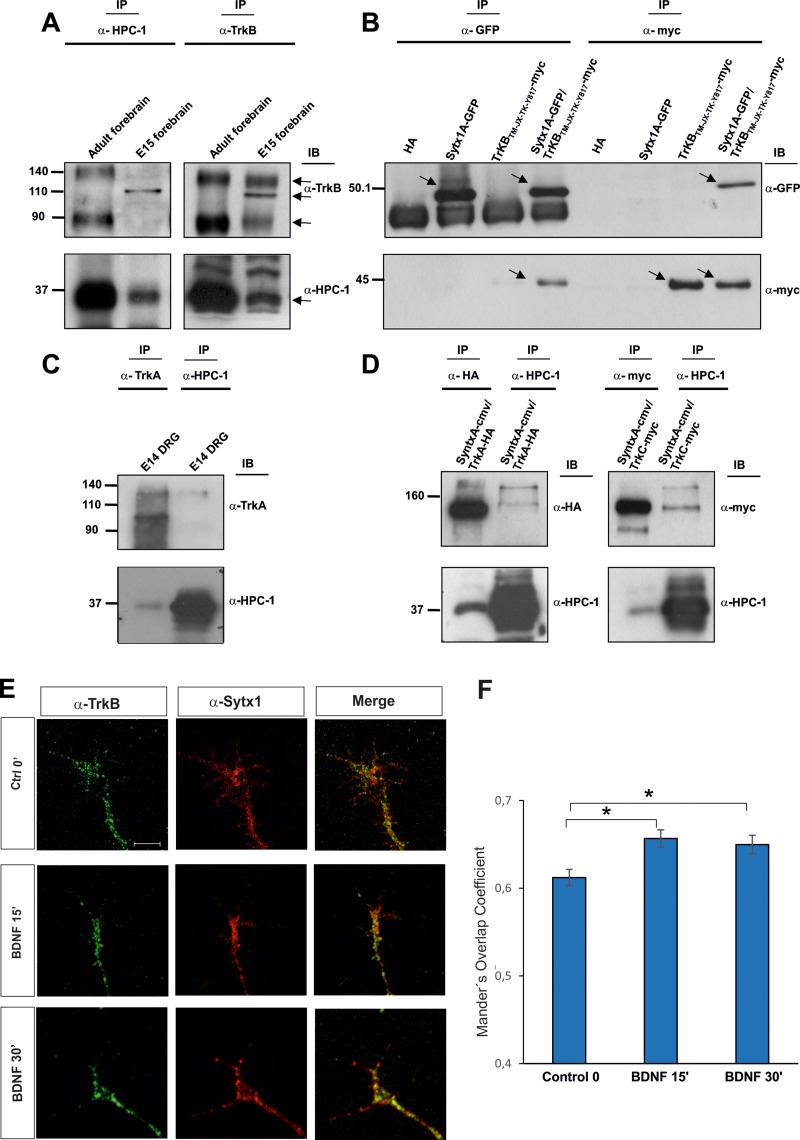
(**A**) TrkB and Sytx1 immunoprecipitation of E15 forebrain and adult homogenates (100 μg of total protein). TrkB immunoprecipitation probed with an anti-Sytx1 antibody (HPC-1) led to co-immunoprecipitation of Sytx1 in both samples. Sytx1 immunoprecipitation caused co-association of TrkB. The TrkB receptor appeared as three bands, consistent with the highly-glycosylated, glycosylated, and unmodified versions of the receptor. For each experiment, three adult brains and pools of E15 embryos of three pregnant mice were used. At least three replicates for each sample were used in experiments. (**B**) Co-immunoprecipitation experiments in HEK293 cells transfected with pSytx1AEGFP alone or together with pCMVtag3A-TrkB_TM-JX-TK-Y817_ tagged with myc. Anti-myc immunoprecipitation resulted in co-association with Sytx1A, visualized with anti-GFP antibodies (lower panel). The reverse immunoprecipitation with anti-GFP antibodies also revealed TrkB protein in the immunoblots with anti-myc (upper panel). (**C**) TrkA and Sytx1 immunoprecipitation of E14 DRG homogenates. TrkA immunoprecipitation probed with an anti-Sytx1 antibody led to co-immunoprecipitation of Sytx1 (lower panel). Sytx1 immunoprecipitation showed co-association of TrkA (upper panel). (**D**) Co-immunoprecipitation experiments in HEK293 cells transfected with Sytx1A-cmv together with TrkA-HA (left panel) and TrkC-myc (right panel). Anti-HA and anti-myc immunoprecipitation resulted in co-association with Sytx1A, as visualized with anti-HPC1 antibodies (lower panels). The reverse immunoprecipitation with anti-HPC1 antibodies also revealed TrkA and TrkC protein in the immunoblots, as visualized with anti-HA and anti-myc antibodies (upper panels). (**E**) Confocal images of hippocampal growth cones treated with BDNF for 0–30 min, immunolabeled for TrkB and Sytx1. Note increased TrkB/Sytx1 co-localization in growth cones incubated with BDNF. An average of thirty growth cones per condition from at least three different experiments were quantified. (**F**) Quantification of TrkB/Sytx1 co-localization signals in hippocampal growth cones expressed as Mander’s overlap coefficient, in cultures treated with BDNF or in control conditions. Arrows indicate specific bands. Significant differences are labeled by asterisks (^*^p ≤ 0.05). Scale bar: E, 3 μm. Error bars indicate SEM.

Neurotrophins bind Trk receptors, thus inducing their dimerization and autophosphorylation at multiple tyrosine residues and leading to the recruitment of various intracellular signaling adaptors and the activation of many signaling pathways [27, 40]. We first studied whether Trk receptors interact with t-SNARE proteins. To this end, we focused on the neurotrophin receptor TrkB because it is the most abundant. To look for a possible interaction between TrkB and proteins of the exocytotic machinery, we performed co-immunoprecipitation analyses in lysates from embryonic (E15) and adult mouse brains. When TrkB antibodies were used to immunoprecipitate brain lysates, a band of approximately 35 kDa was detected by Western blot (WB) with the HPC-1 antibody, which recognizes Sytx1. Furthermore, in immunoblots of brain lysates immunoprecipitated with anti-Sytx1 antibodies, TrkB bands of 140 and 90 kDa were identified in adults, while a TrkB band of 110 kDa was detected in E15 extracts (Figure 1A, Supplementary Figure 1). These three bands are likely to correspond to differentially glycosylated TrkB receptors. To corroborate these results, we co-immunoprecipitated HEK293 cells co-transfected with DNAs encoding for both proteins, namely TrkB_TM-JX-TK-Y817_ (tagged with myc) and Sytx1AEGFP. In lysates immunoprecipitated with anti-myc antibodies, Sytx1AEGFP was detected by WB using anti-GFP. Pull-downs with anti-GFP antibodies revealed TrkB protein. No co-immunoprecipitation was detected when cells were transfected with Sytx1AEGFP or TrkB_TM-JX-TK-Y817_ DNA alone (Figure 1B, Supplementary Figure 1).

Next, we explored whether the TrkA receptor also interacts with SNARE proteins. In embryonic DRGs, which express high TrkA levels, immunoprecipitation of TrkA showed Sytx1 co-association. Similarly, immunoprecipitation with anti-Sytx1 antibodies yielded visualization of the full-length TrkA form by immunoblotting (Figure 1C).

As a further step, we performed immunoprecipitation experiments *in vitro* using TrkA and TrkC constructs. We co-immunoprecipitated HEK293 cells co-transfected with DNAs encoding for both proteins (TrkA-HA or TrkC-myc with Sytx1A-CMV). In lysates immunoprecipitated with anti-HA antibodies, Sytx1A-CMV was detected by WB using anti-HPC1. Pull-downs with anti-HPC1 antibodies revealed TrkA protein (Figure 1D, left panel). In lysates immunoprecipitated with anti-myc antibodies, Sytx1A was detected by WB using anti-HPC1. Pull-downs with anti-HPC1 antibodies revealed TrkC protein (Figure 1D, right panel). Together, these data indicate that Sytx1A co-associates with TrkA, TrkB and TrkC after expression in non-neuronal cells.

To determine whether neurotrophins regulate the association of TrkB and Sytx1, we incubated hippocampal cultures with BDNF and then measured overlapping signals in axonal growth cones by immunofluorescence. A significant increment in Sytx1/TrkB co-localization was detected in these structures after 15 and 30 min of treatment with BDNF (Figure 1E, 1F). These data suggest that BDNF increases the co-association of TrkB receptor and Sytx1 in the growth cones of developing neurons. (Figure 1E, 1F).

### Interaction with other SNARE proteins

**Figure 2:**
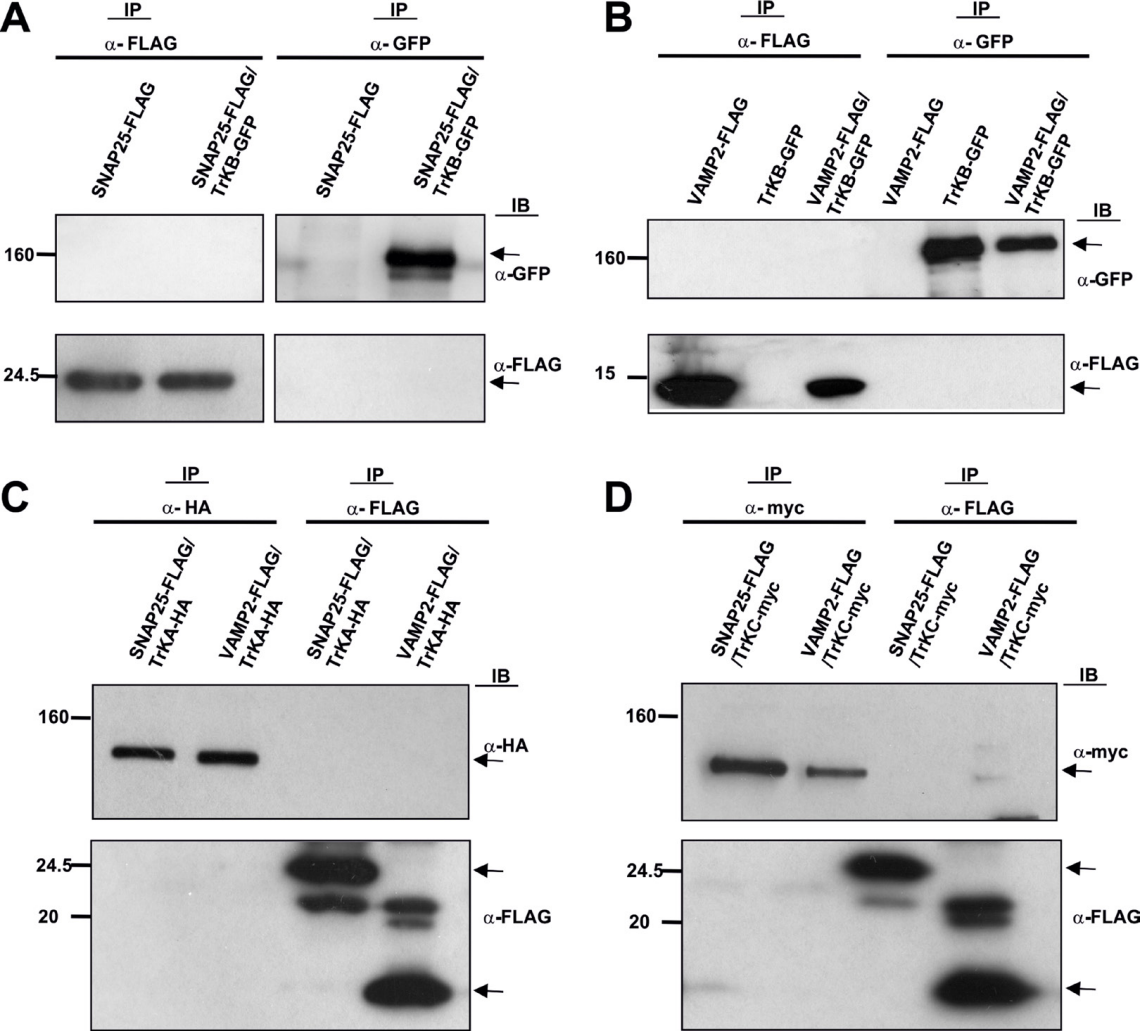
Co-immunoprecipitation experiments in HEK293 cells transfected with (**A**) pEF-BOS-SNAP25-FLAG alone or together with pEGFPC1-TrkB. (**B**) pEF-BOS-VAMP2-FLAG alone or together with pEGFPC1-TrkB. (**C**) pEF-BOS-SNAP25-FLAG, pEF-BOS-VAMP2-FLAG and TrkA-HA. (**D**) pEF-BOS-SNAP25-FLAG, pEF-BOS-VAMP2-FLAG and TrkC-myc. No co-immunoprecipitation was observed between proteins analyzed with anti α-GFP, anti-myc, anti-FLAG, or anti-HA antibodies. Two transfections in HEK cells per condition were run in parallel for each experiment and three experiments were done. Arrows indicate specific bands.

Since Trk receptors interact with Sytx1, we reasoned that they might associate with additional SNARE proteins. Negative co-immunoprecipitation results were observed when TrkB, SNAP25 (pEF-BOS-SNAP25-FLAG and pEGFPC1-TrkB) (Figure 2A, Supplementary Figure 1), and VAMP2 (pEF-BOS-VAMP2-FLAG and pEGFPC1-TrkB) (Figure 2B, Supplementary Figure 1) were overexpressed in HEK293 cells. To address whether the above pattern of interactions was also common to other Trk receptors, we studied the interaction between TrkA or TrkC and SNARE proteins. Negative co-immunoprecipitation was observed again when TrkA or TrkC were co-transfected with SNAP25 and VAMP2 (Figure 2C, 2D) in these cells.

To confirm these findings, a series of co-immunoprecipitation analyses were also performed in lysates from embryonic (E15) and adult brains. We did not detect co-immunoprecipitation of TrkB with either SNAP25 or VAMP2. This observation was then confirmed by immunoprecipitating VAMP2 and SNAP25 and immunoblotting with anti-TrkB (Supplementary Figure 2).

**Figure 3:**
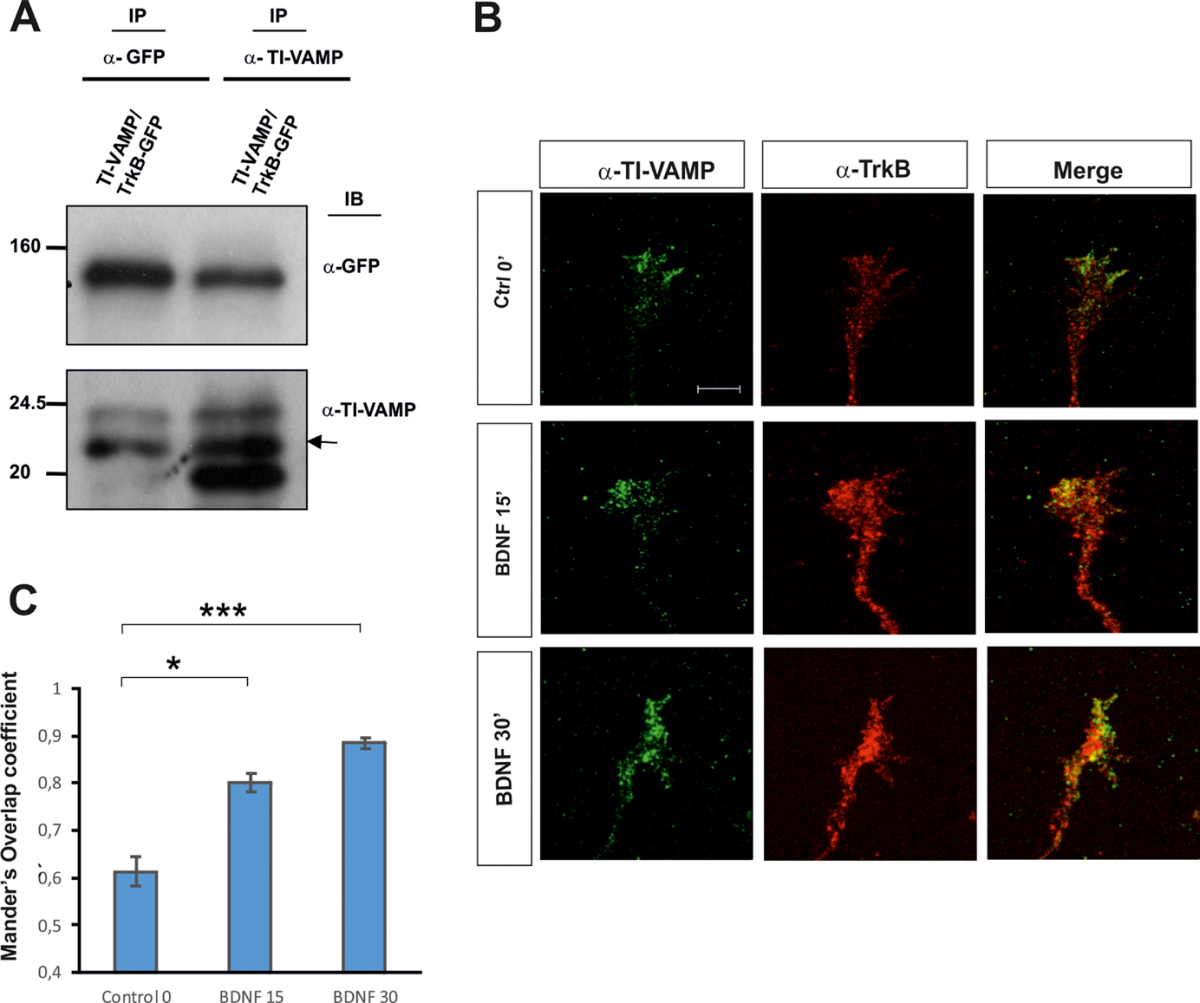
(**A**) Co-immunoprecipitation experiments in HEK293 cells transfected with TrkB-GFP and TI-VAMP showing an *in vitro* interaction. Two transfections in HEK cells per condition were run in parallel for each experiment and three experiments were done. (**B**) Confocal images of hippocampal growth cones treated with BDNF for 0–30 min, immunolabeled for TrkB and TI-VAMP. Note increased TrkB/TI-VAMP co-localization in growth cones incubated with BDNF. (**C**) Quantification of TrkB/ TI-VAMP co-localization signals in hippocampal growth cones expressed as Mander’s overlap coefficient, in cultures treated with BDNF or in control conditions. An average of twenty growth cones per condition were quantified in four experiments using different cell cultures. Significant differences are labeled by asterisks (^*^p ≤ 0.05; ^***^p ≤ 0.001). Scale bar: B, 3 μm. Error bars indicate SEM.

As the formation of the SNARE complex involves v-SNAREs and t-SNAREs, we next studied the possible interaction between the t-SNARE TI-VAMP and the Trk receptor. We co-immunoprecipitated HEK293 cells cotransfected with DNAs encoding for TrkB-GFP and TI-VAMP. In lysates immunoprecipitated with anti-GFP, TI-VAMP was identified by WB using anti-TI-VAMP (Figure 3A). The reverse immunoprecipitation assay also indicated a TI-VAMP/TrkB interaction in transfected cells (Figure 3A).

Additionally, we treated hippocampal primary cultures with BDNF for 0, 15 and 30 min and analyzed the co-localization between TrkB and TI-VAMP. Confocal images and intensity correlation analysis indicated an increase in the co-localization of both proteins with respect to the control (Figure 3B, 3C). Together, these results may suggest that TI-VAMP, like Sytx-1, interacts with TrkB receptors in a BDNF-dependent manner.

### Identification of the protein region responsible for Trk/Sytx1 interaction

**Figure 4:**
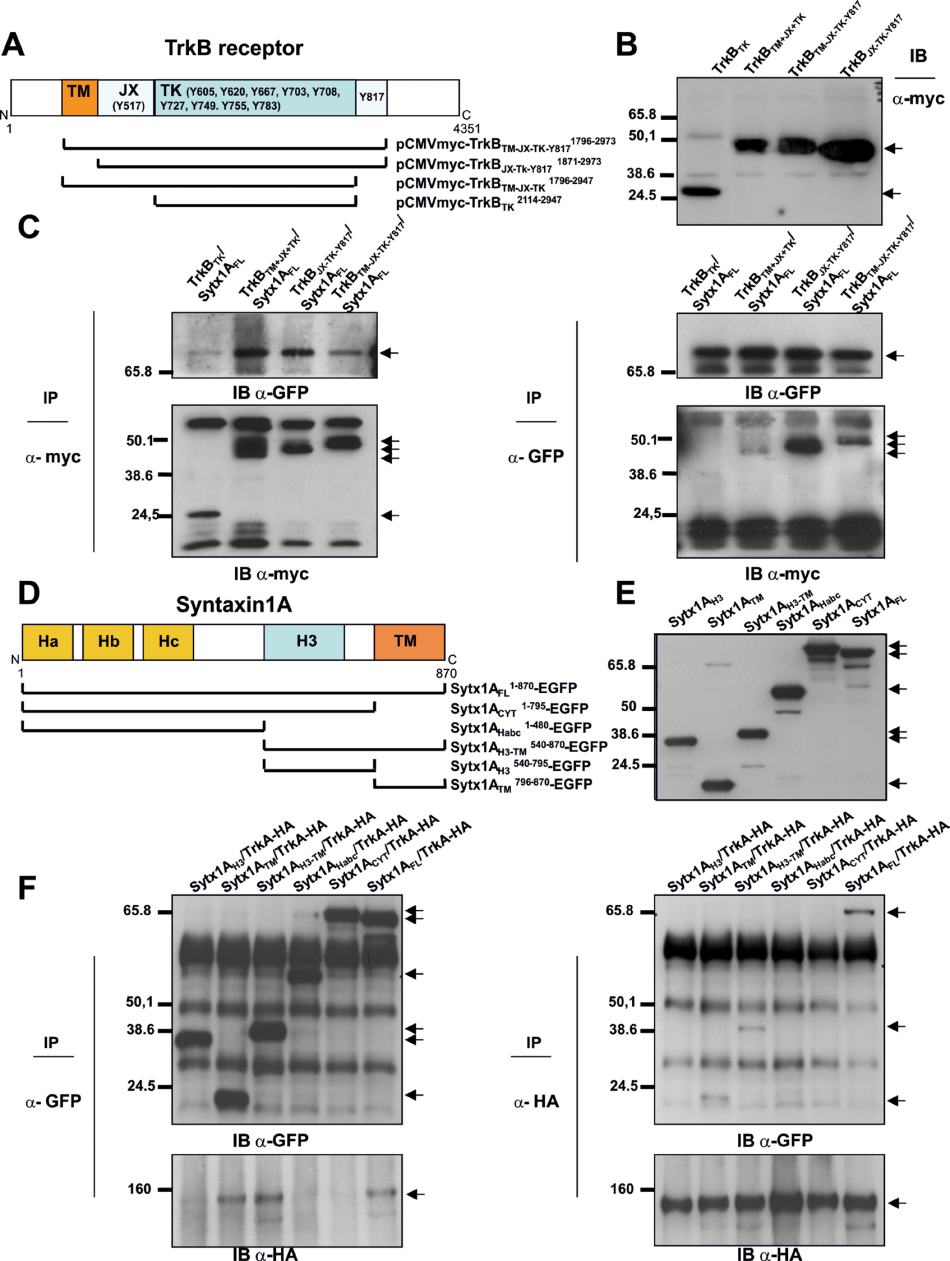
Characterization of protein regions required for Sytx1A/DCC interaction (**A**) Diagram summarizing TrkB domains and the truncated TrkB-myc chimeras generated. (**B**) Western blot for TrkB fragments: expression of the truncated TrkB-myc DNAs in HEK293 cells, showing that they generate proteins of the appropriate molecular weight, between 24 and 50 kDa, as revealed by immunoblotting with anti-myc antibodies (**C**) Myc antibodies co-immunoprecipitated Sytx1-GFP (left panel, arrow). The reverse experiments, using anti-GFP antibodies to immunoprecipitate Sytx1A, and immunoblotting with anti-myc antibodies yielded identical results as above (right panel, arrows). (**D**) Diagram summarizing Sytx1A domains and the truncated Sytx1AEGFP chimeras generated. (**E**) Expression of the several Sytx1AEGFP DNAs in HEK293 cells, showing that they generate proteins of the appropriate molecular weight, between 65 and 27 kDa, as revealed by immunoblotting with anti-GFP antibodies. (**F**) Western blot analysis of transfected cell with Sytx1 constructs and TrkA-HA. Co-transfection with either the Sytx1A_CYT_EGFP or the Sytx1A_Habc_EGFP constructs did not reveal co-association. In contrast, HA antibodies co-immunoprecipitated the Sytx1A_H3TM_EGFP (right panel, upper). The reverse experiments, using anti-GFP antibodies to immunoprecipitate EGFP-tagged Sytx1A chimeras, and immunoblotting with anti-HA antibodies gave identical results as above (left panel, bottom). Arrows indicate specific bands.

To identify the domains of TrkB necessary for its interaction with Sytx1EGFP, we generated four truncated TrkB proteins in the pCMVtag3a-Myc vector. There are 10 known conserved tyrosine residues in the cytosolic tail of mammalian Trk receptors, [41, 42]. The Y670, Y674 and Y675 sites in the TrkA receptor are located in the auto-regulatory loop of the tyrosine kinase domain and thus modulate kinase activity when phosphorylated. Other tyrosines are responsible for the interaction with adaptor and scaffold proteins that contain phosphotyrosine binding (PTB) domains or Src-homology-2 (SH2) domains. The best characterized of these docking sites in human TrkA are Y490 and Y785. We thus generated the following constructs: the tyrosine kinase domain, containing the three tyrosine residues of the auto-regulatory loop of TrkB (TrkB_TK_); the transmembrane-juxtamembrane-tyrosine kinase domain, carrying the analogous tyrosine 490 (Y490) in TrkB receptors (TrkB_TM-JX-TK_); the juxtamembrane-tyrosine kinase domain, lacking the transmembrane domain (TrkB_JX-TK-Y817_) but including the Y817 analogous to the Y785 of TrkA receptors; and the entire intracellular domain, which carries all the phosphorylatable catalytic tyrosine residues, which included the transmembrane domain (TrkB_TM-JX-TK-Y817_) (Figure 4A). DNAs were expressed in HEK293 cells, and their expression levels and predicted molecular weights were measured by immunoblot (Figure 4B). TrkB DNA constructs were transfected in combination with Sytx1A, and lysates were processed for immunoprecipitation and immunoblotting. Co-immunoprecipitation with GFP antibodies revealed co-association of Sytx1A with all four constructs (Figure 4C, right panel). The reverse experiments using anti-myc antibodies to immunoprecipitate the TrkB fragments confirmed this result (Figure 4C, left panel). These findings indicate that the TK fragment is the basic region necessary for the interaction with Stx1.

We next attempted to identify the domains of Sytx1A required for its interaction with Trk receptors. We generated several truncated Sytx1A constructs to which EGFP was fused at the N-terminus region (Cotrufo et al., 2011). We used six constructs carrying the H3 (Sytx1A_H3_), transmembrane (Sytx1A_TM_), H3-transmembrane (Sytx1A_H3TM_), and the Habc (Sytx1A_Habc_) domains, as well as the complete cytosolic tail (Sytx1A_CYT_) and a full-length construct (Sytx1A_FL_) (Figure 4D). The predicted molecular weights were measured by immunoblot (Figure 4E). Constructs were transfected in combination with pCDNA-TrkA-HA (Figure 4F). Co-transfection with either the Sytx1A_CYT_EGFP, Sytx1A_H3_ or Sytx1A_Habc_EGFP constructs did not show co-association. In contrast, HA antibodies co-immunoprecipitated the Sytx1A_H3TM_EGFP and Sytx1A_TM_EGFP chimeras (Figure 4F, left panel). The reverse experiments, using anti-GFP antibodies to immunoprecipitate EGFP-tagged Sytx1A chimeras and immunoblotting with anti-HA antibodies, yielded results identical to those above (Figure 4F, right panel). Sytx1A and TrkB appeared to co-associate through the TM region of Sytx1A and the auto-regulatory loop containing the tyrosine kinase domain of TrkB.

### Botulinum toxin C1 blocks axonal growth of Nodose and DRG axons

The sensory neurons of the Nodose ganglia are a classic example of peripheral nervous system neurons that do not require NGF for survival or outgrowth during development. However, they are dependent on BDNF and to a lesser extent on NT-3 and NT-4 [43, 44]. In the dorsal root ganglion (DRG) and trigeminal ganglia, a large population of neurons conveying different modalities of sensory information express TrkA, and the viability and outgrowth of these cells depends on NGF [45]. Nevertheless, other DRG neurons depend on NT-3 and TrkC [46, 47]. Therefore, we used Nodose ganglia and DRGs as models to study the effect of Sytx1 blockade on TrkB-, TrkC- and TrkA-expressing populations, respectively. The specific response of these populations to BDNF, NT-3 and NGF allowed us to study the effect of Botulinum toxins on axonal outgrowth. Botulinum toxins are metallo-proteases that markedly reduce the exocytosis of synaptic vesicles and neurotransmitter release by cleaving specific SNARE proteins [48]. While Botulinum Toxin A (BoNT/A) cleaves only SNAP25, Botulinum Toxin C1 (BoNT/C1) cleaves both Sytx1 and SNAP25, and Clostridium tetanus toxin (TeNT) cleaves VAMP2 [49, 50].

**Figure 5:**
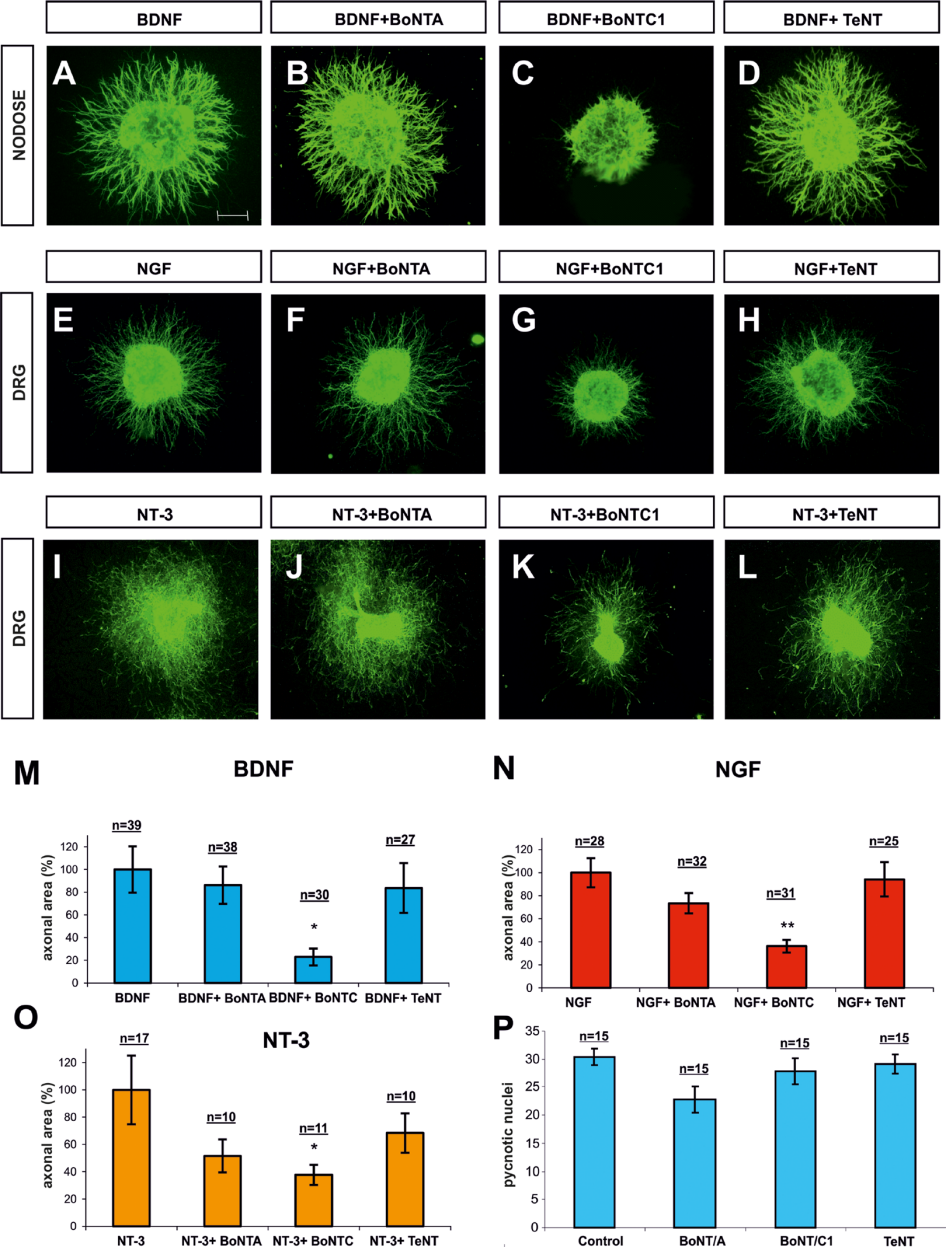
Confocal images of Nodose and DRG explants co-cultured in collagen gel Explants were immunolabeled for βIII-Tubulin and counterstained with fluorescent anti-mouse 488. Explants were co-cultured with BDNF (**A**–**D**), NGF (**E**–**H**) and NT3 (**I**–**L**). In each row, explants were treated with BoNT/A, BoNT/C1 and TeNT. Bar graph expressing the area of neurites in Nodose explants supplemented with BDNF (**M**) and in DRG explants supplemented with, NGF (**N**) and NT-3 (**O**), and treated with BoNT/A, BoNT/C1 and TeNT. Bar graph showing the number of cells with pycnotic nuclei, indicating that toxin treatment did not affect neuronal death (**P**). N indicates the number of explants obtained from different animals in different experiments. Nodose explants came from five experiments in which at least three embryos per condition were used. DRG explants were obtained from six experiments in which one embryo per condition was used. Significant differences are labeled by asterisks (^*^p ≤ 0.05; ^**^p ≤ 0.01). Scale bar: A, 300 μm. Error bars indicate SEM.

Nodose (Figure 5A–5D) and DRG (Figure 5E–5L) explants cultured in collagen gels were supplemented with BDNF, NGF and NT-3 (all 50 ng/ml). Explants were cultured for 1 day in the presence of 15 nM BoNT/C1, 25 nM BoNT/A or 2nM TeNT, and axonal growth in the presence and absence of toxins was compared. Nodose and DRG explants, incubated with BDNF or NGF respectively, did not show a significant decrease in axonal growth when cultured with BoNT/A or TeNT (Figure 5B, 5F, 5J, 5D, 5H and 5L). On the other hand, those treated with BoNT/C1 showed high inhibition of axonal growth (BDNF, NGF) (Figure 5C, 5G and 5K, 5M, 5N). A similar scenario was observed when DRG explants were incubated with NT3, in which case a statistically significant decrease was also observed in explants incubated with BonT/C1 but not with BonT/A and TeNT (Figure 5I–5L). Finally, we counted the number of pyknotic nuclei present in Nodose ganglia incubated with toxins. Treatment with BoNT/C1, BoNT/A and TeNT did not result in an increased number of pyknotic cells, thereby indicating that in our experimental conditions Botulinum toxins did not lead to neuronal cell death (Figure 5P). We thus conclude that cleavage of Sytx1, but not of SNAP25 or VAMP2, inhibits neurotrophin-dependent axonal growth. This finding, together with the observation of a specific interaction between Trk receptors and Sytx1, suggests that this SNARE protein is required for neurotrophin-mediated neurite extension and outgrowth.

**Figure 6:**
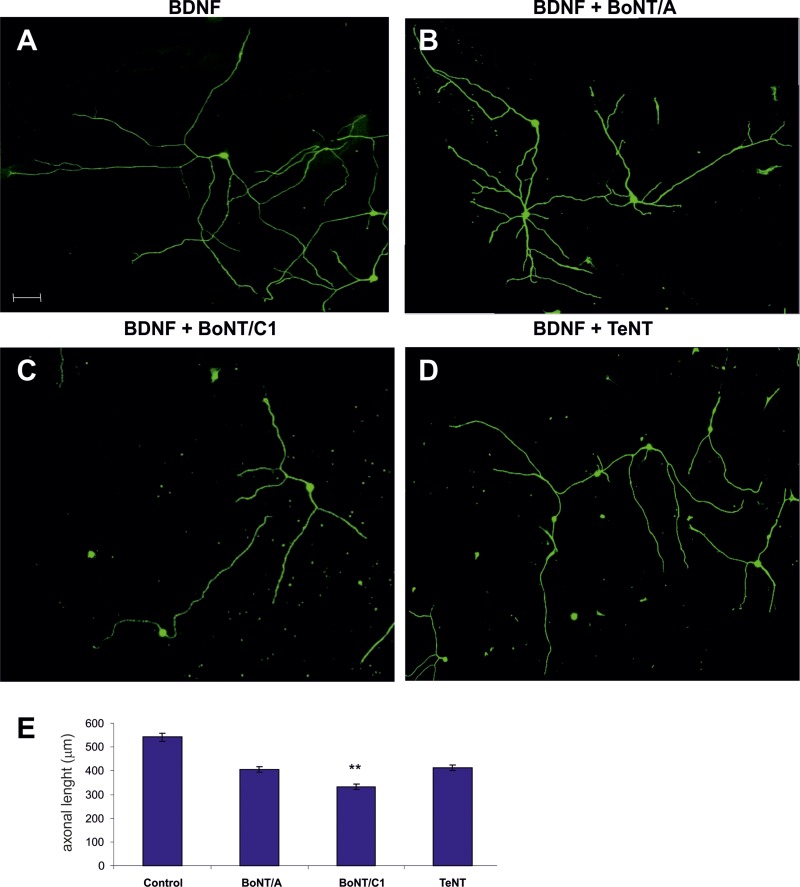
Examples of Nodose primary cultures supplemented with BDNF (**A**) and treated with BoNT/A (**B**), BoNT/C1 (**C**) and TeNT (**D**–**E**) Bar graph showing the quantification of neurite length when treated with neurotoxins compared with control. Significant differences are labeled by asterisks (^**^p ≤ 0.01). An average of thirty neurons per condition were quantified in five separate experiments. Scale bar: A, 50 μm. Error bars indicate SEM.

To corroborate these data, we next used dissociated primary Nodose cultures supplemented with BDNF and treated them with BoNT/C1, BoNT/A and TeNT (Figure 6). Individual neurite length was measured. A significant decrease in this parameter was found only in cultures incubated with BoNT/C1 (Figure 6E).

### Neurons require Sytx1 and TI-VAMP for BDNF-dependent outgrowth

**Figure 7:**
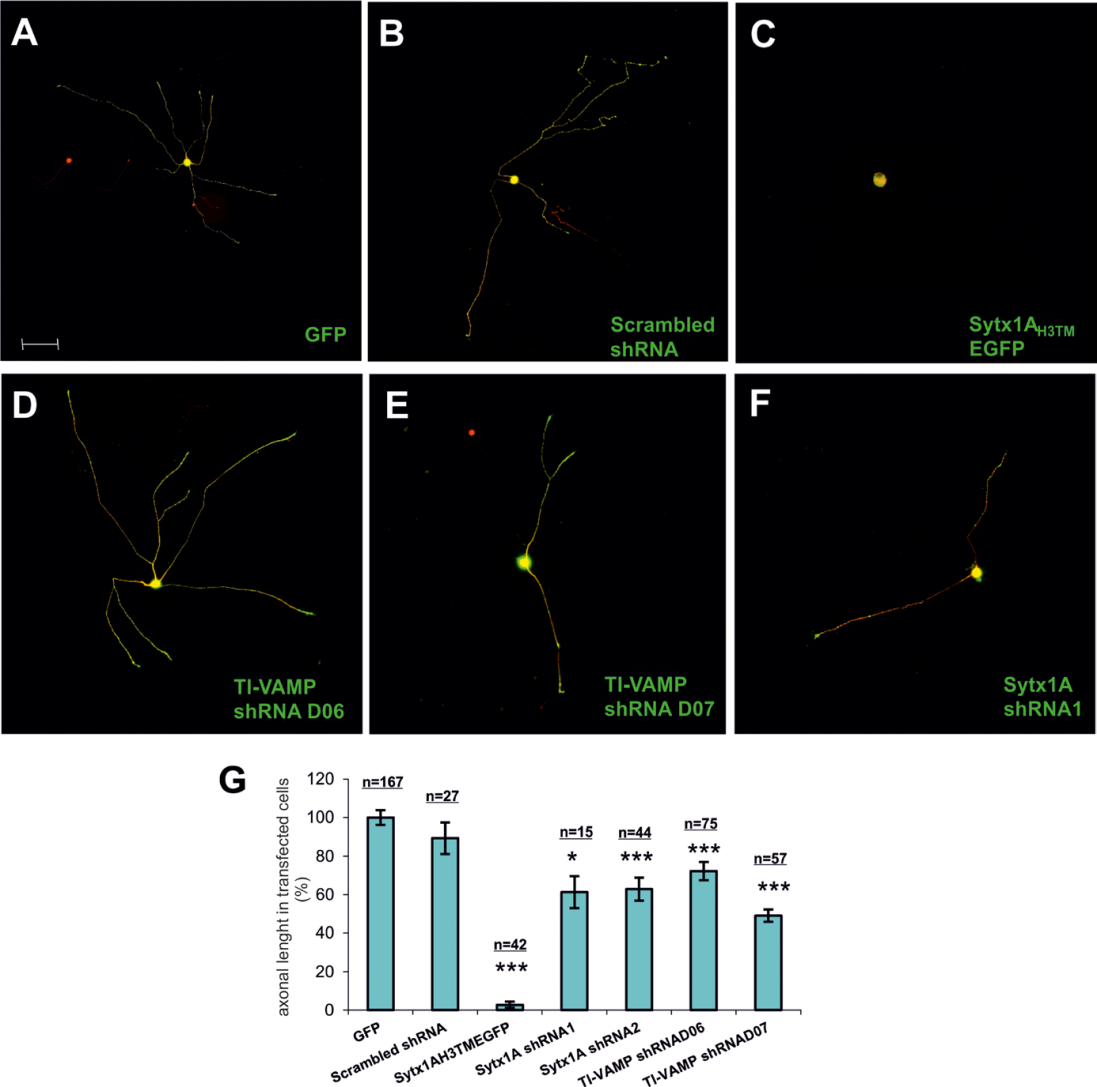
Examples of electroporated Nodose primary cultures supplemented with BDNF. Cells were electroporated with (**A**) GFP, (**B**) scrambled shRNA, (**C**) SytxH3TMEGFP, (**D**) TI-VAMP shRNAD06, (**E**) TI-VAMP shRNAD07, and (**F**) Sytx1A shRNA1. Cells were immunolabeled for βIII-Tubulin followed with secondary anti-mouse coupled to alexafluor 568 and counterstained with anti-GFP followed by fluorescent anti-rabbit 488. (**G**) Bar graph representing the percentage of transfected axonal length normalized to the control condition. An average of eighty neurons per condition were quantified in four experiments using different cell cultures. Significant differences are labeled by asterisks (^*^p ≤ 0.05; ^***^p ≤ 0.001), (n indicates the number of neurons analyzed per condition). Scale bar A, 50. Error bars indicate SEM.

To further substantiate the notion that the TrkB- Sytx1 interaction is required for BDNF-dependent axonal growth, we generated a Sytx1A_H3TM_ construct containing the minimal Sytx1A interaction domain, as a dominant negative to interfere with Sytx1 (Cotrufo et al., 2011). We cloned Sytx1A_H3TM_ into a pIRES-EGFP vector (Clontech) to generate a unique transcript capable of translating both target and EGFP proteins. We also took advantage of two shRNAs for TI-VAMP (D06 and D07) and two shRNAs for Sytx1A (shRNA1 and shRNA2). The shRNA sequences were tested in transfected cells to assess the percentage of silencing. D06 TI-VAMP shRNA induced a 50% decrease in expression while D07 TI-VAMP shRNA achieved about 85% (Supplementary Figure 3). Nodose primary cultures were then electroporated with control pIRES-EGFP (Figure 7A), pIRES-Sytx1A_H3TM_EGFP (Figure 7C), TI-VAMP (Figure 7D, 7E), Sytx1 shRNAs (Figure 7F), and a scrambled shRNA sequence (SCR) (Figure 7B). Finally, we treated the cultures with BDNF (50 ng/ml) for one day. We then labeled them with βIII-Tubulin and anti-GFP antibodies (Figure 7). Interfering with Sytx1A or TI-VAMP siRNA caused a significant reduction in axonal length with respect to control (scrambled and EGFP) electroporations. Moreover, electroporation with Sytx1A_H3TM_EGFP markedly reduced this parameter (about 95%). Together, these findings indicate that interference with Sytx1 function via expression of a Sytx1A_H3TM_ dominant negative or using siRNAs blocks BDNF-mediated axonal growth in dissociated neurons (Figure 7G).

### BDNF-mediated exocytosis in Nodose growth cones

The results presented here point to a biochemical and functional link between Trk receptors and the proteins involved in exocytotic machinery. Additional studies were done to corroborate this hypothesis. Bodipy-ceramide has been used to study vesicle formation and exocytosis in non-neuronal and neuronal cells [40, 51]. Bodipy-ceramide has a concentration-dependent emission spectrum, with a maximum at 515 nm (green) at low concentration. In the growth cone, green label was evident in the plasma membrane, extending into the filopodia and lamellipodia. At high concentration, Bodipy-ceramide formed excimers with an emission maximum at 620 nm (red). These features allow selective visualization of Golgi-derived vesicles (those secreted during exocytosis). The presence of Bodipy-ceramide labeling along neurites was also evident as dot-like structures that accumulated at growth cones. As suggested by Huber and colleagues, it is likely that the dots represent exocytic vesicles in transit to neuritic tips, where new membrane addition takes place [52].

**Figure 8:**
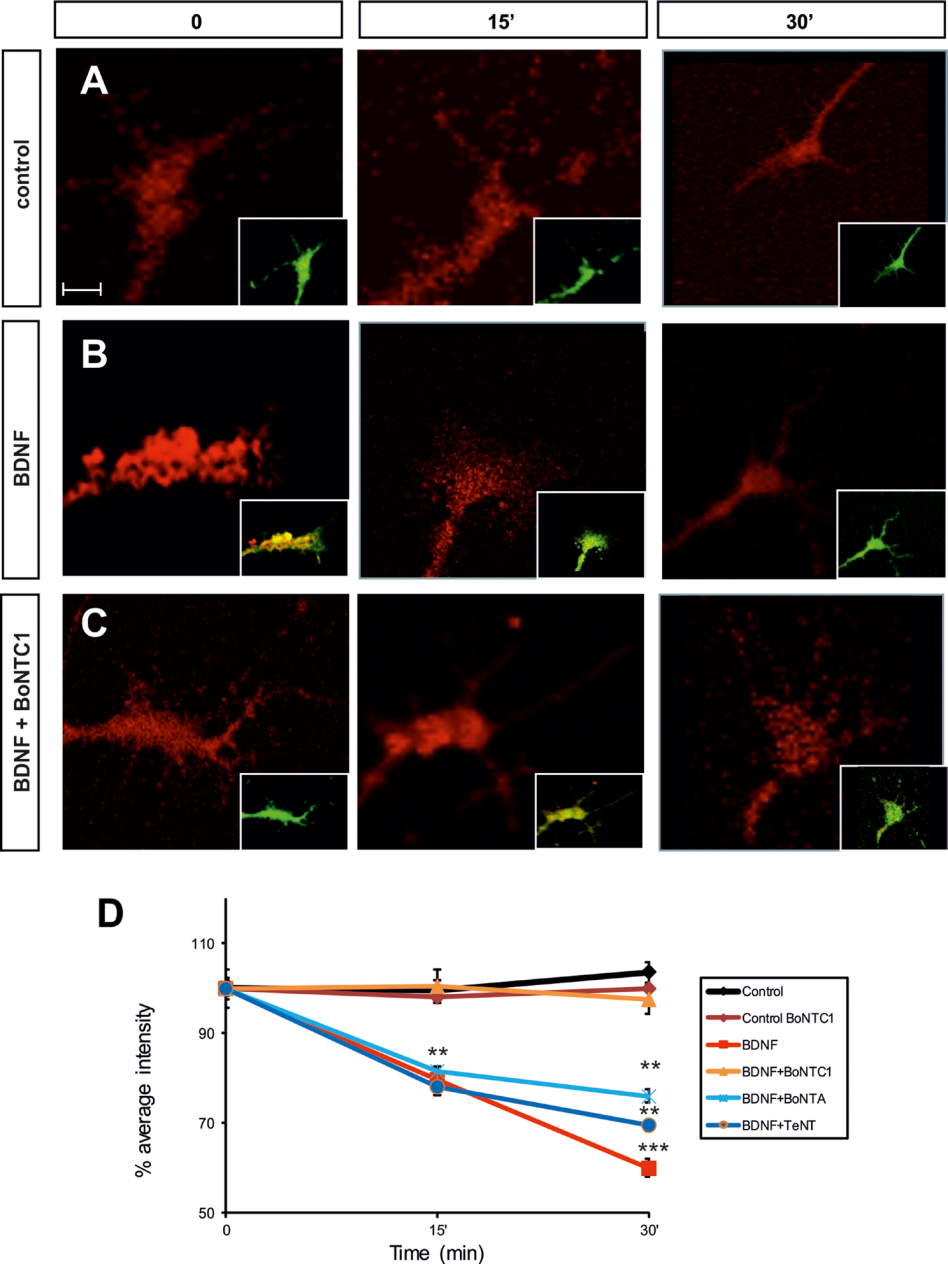
Fluorescence micrographs of the Nodose explant axonal growth cones Neurons were labeled with Bodipy-ceramide. Growth cones were challenged with control medium (**A**), BDNF (**B**), after factor deprivation, or BDNF+BoNT/C1 (**C**), for the time indicated in minutes. Percentage of average intensity of red fluorescent puncta in growth cones for control, BDNF-, BoNT/C1-, BoNT/A- and TeNT-treated Nodose neurons (normalized to 100% at the onset of treatment) (**D**). Data points are means ± s.e.m. from eighty growth cones quantified from an average of eighteen explants per condition taken from six embryos in three separate experiments. Significant differences are labeled by asterisks (^**^p ≤ 0.01; ^***^p ≤ 0.001). Scale bar: A, 2 μm. Error bars indicate SEM.

To study whether BDNF/TrkB regulates exocytosis in a SNARE-dependent manner, we pulse-labeled Nodose explants with Bodipy-ceramide at room temperature and then chased them for 2.5–3 h at 37°C. Hence, all membrane addition experiments were recorded in the green (cytoplasmatic membrane) and red channels (exocytosis vesicles). The results of these pulse–chase experiments revealed an initial accumulation of Bodipy-ceramide label (red channel) in the Golgi apparatus, followed by the sequential appearance of vesicle-like structures in the cell body, neurites, and growth cones (Figure 8A–8C). We quantified the average pixel ratio (red/green), which gave a direct measurement of the red spots at the growth cones (Figure 8D). Incubation with BDNF led to a marked reduction of Bodipy-ceramide fluorescence between 15 and 30 min, thereby indicating depletion and release of pre-labeled vesicles (Figure 8B, 8D). Conversely, BDNF and BoNT/C1 treatment showed a constant level of fluorescence over time, indicating the inhibition of exocytosis (Figure 8C, 8D).

In order to corroborate that VAMP2 and SNAP25 were not involved in this mechanism, we stimulated Nodose neurons with BDNF and simultaneously treated them with BoNT/A and TeNT. As in BDNF treatment, fluorescence decreased at 15 and 30 min, thereby demonstrating the fusion of exocytotic vesicles at the membrane (Figure 8D).

These results indicate that Sytx1 cleavage abolishes the exocytosis of pre-labeled vesicles and demonstrate that Sytx1 is required for BDNF-regulated exocytosis in the growth cone.

## DISCUSSION

Axonal growth is a complex event in which the mechanisms that regulate elongation, fasciculation and guidance are closely coordinated. During pathfinding, growth cones exhibit a variety of behaviors, such as cone collapse, repulsion and attraction [41, 53–57]. Such activities involve cytoskeletal re-arrangements and plasmalemma re-modeling [4, 10, 13, 58]. In this context, neurotrophins play an important role as trophic molecules, regulating cell death and proliferation [23, 32, 59], as well as neurite extension and axonal guidance [31, 32]. The activation of neurotrophin receptor Trk has been shown to result in the induction/triggering of several signaling pathways that play instructive roles in cytoskeleton regulation [60], membrane expansion [61], gene transcription [62, 63], and protein translation [64, 65].

The addition of new membrane to nerve processes is an essential step in nerve growth and differentiation and also in axonal guidance [13, 66, 67]. We have already described that Netrin-1 receptor DCC interacts with Sytx1 in a Netrin-1-dependent manner to exert chemoattraction and to guide axons and migrating neurons toward their final destination [17, 18]. In the present study, we addressed whether other key secreted molecules, namely neurotrophins, also require SNARE proteins to exert their functions during development. To this end, we first focused on the interaction between neurotrophin receptors with classical SNARE complex proteins. As the TrkB receptor is the most widely expressed receptor in the CNS, we examined whether TrkB and Sytx1 interact in adult and embryonic forebrains. We demonstrated that TrkB receptors interact *in vivo* and *in vitro* with Sytx1, but not with VAMP2 or SNAP25. Furthermore, co-immunoprecipitation assays *in vitro* revealed that TrkA and TrkC also interact with Sytx1, thereby supporting the notion of a common relationship between Trk receptors and the proteins regulating exocytosis. The interaction of Trk receptors with Sytx1 was confirmed by co-immunoprecipitation assays using specific domains of these proteins. Our results also suggest that this interaction is regulated by Trk ligands, since BDNF treatment of hippocampal growth cones increased TrkB/Sytx1 co-localization. Thus, together with our previous findings on the Netrin-1 receptor [17, 18] and other studies reporting interactions of other guidance receptors with SNARE proteins [13, 68, 69], our findings support the notion of a common mechanism for receptors that regulate guidance and neurite extension. In this mechanism, the receptors are coupled to SNARE proteins in order to mediate the guidance and outgrowth roles of the former, thereby revealing a tight regulation of exocytosis by the activation of guidance receptors [70]. However, we cannot exclude that Sytx1 exerts a different role in neurotrophin signaling, since in unstimulated conditions the degree of TrkB/Sytx1 co-localization was relatively high. We found that Trk receptors did not co-associate with SNAP25 or VAMP2. Previous experiments showed that SNAP25 is essential for evoked synaptic transmission, but not for nerve growth or stimulus-independent neurotransmitter release [71]. In addition, in SNAP25 null mutants, SNAP24 exerts its function in neurotransmitter release [72]. With regard to VAMP2, cleavage of VAMP2 by TeNT or BoNT/B has no effect on neurite extension or synaptogenesis [73]. Similar results were found in VAMP2 KO mice [74]. VAMP2 may function by catalyzing fusion reactions and stabilizing fusion intermediates; however, it is not required for synaptic fusion [74].

Exocytosis requires at least one v-SNARE protein, in addition to Sytx1. In previous work, we found that DCC receptors interact with TI-VAMP in a Netrin-dependent manner to induce exocytosis in growth cones [18]. Therefore, here we studied the interaction between Trk receptors and TI-VAMP protein and its dependence on BDNF. Trk receptors interacted with the developmentally regulated and growth cone-enriched v-SNARE TI-VAMP. We used clostridial neurotoxins to examine the function of SNARE proteins, as the former cleave the latter in a specific way [48–50], and peripheral ganglia as a model to study the different neurotrophin-dependent neuronal populations. Our results showed that, in Nodose and DRG explants and in primary cultures, the cleavage of Sytx1 by BoNT/C1 decreases BDNF-, NGF- and NT3-dependent axonal growth (Figures 5, 6). In contrast, cleavage of SNAP25 and VAMP2 by BoNT/A and TeNT did not affect NGF-dependent or NT3-dependent axonal growth. These results indicate that Sytx1, but not SNAP25 or VAMP2, is important for neurotrophin-mediated axonal growth. These results are in agreement with the above co-immunoprecipitation experiments, which demonstrated the interaction of Trk receptors with Sytx1, but not with SNAP25 or VAMP2. On the other hand, we found that BoNT/C1 treatment of dissociated Nodose neurons resulted in a decrease in neurite length. These results were confirmed by experiments using the Sytx1-H3TM fragment (as a Sytx1 negative dominant), as well as siRNA downregulation strategies. Sytx1 and TI-VAMP downregulation, as well as the expression of the Sytx1-H3TM dominant negative, dramatically reduced neurite length in ganglia and hippocampal neurons treated with BDNF. These findings support the hypothesis that the interaction between TrkB and Sytx1 is necessary for BDNF-mediated axonal growth and that a functional TI-VAMP/Sytx1 complex is required for BDNF-mediated axonal growth during development.

The previous results point to a biochemical and functional link between the receptors for trophic factors and the proteins that regulate exocytosis. We demonstrate that treatment with BoNT/C1 reduces axonal elongation in Nodose and DRG explants, as a result of the cleavage of Sytx1. Finally, we sought to address how SNARE proteins allow the elongation of neuronal axons by an exocytotic process. To this end, we used Bodipy-ceramide to explore exocytosis induction during BDNF-dependent axonal growth in Nodose explants. Incubation with BDNF led to the release of pre-labeled vesicles at the cone tips (Figure 8). Additionally, treatment with BoNT/C1 indicated that Sytx1 cleavage abolished the decrease in fluorescence and therefore the exocytosis of pre-labeled vesicles (Figure 8). Nevertheless, treatments with BoNT/A and TeNT did not change the rate of exocytosis. These results suggest that Sytx1 is required for BDNF-regulated exocytosis in growth cones.

Our results might also explain why Sytx1 is an absolute requirement for neuronal survival and maintenance [75]. Recent articles outline the importance of Sytx1 in these processes [42]. Although Syntaxin relevance has been ascribed to a lack of trophic supply by glia, the growth of Sytx1A/1B double knock-out hippocampal neurons on wild-type astrocytes still impaired their survival, thereby indicating a cell-autonomous effect. The present study, showing a Sytx1-dependent regulation of Trk receptor function, could fill this gap.

In conclusion, our results demonstrate that neurotrophin/Trk receptor signaling requires two SNARE proteins, namely Sytx1 and TI-VAMP, to mediate axonal outgrowth and extension. Together with previous studies showing that various SNARE complex proteins are necessary for the signaling of guidance molecules exerting either chemoattraction (e.g. Netrin1, [17, 18]) or chemorepulsion (e.g., class III Semaphorins, [69]; Slits/Robo [76], our findings on neurotrophins—another family of extracellular factors that mediates axonal growth—support the notion that the association of receptor complexes with specific sets of SNARE proteins that mediate exocytosis or endocytosis is a general mechanism by which neurotrophic factors and guidance cues exert their effects to promote axonal guidance and growth.

## MATERIALS AND METHODS

### Animals

OF1 embryos and adult mice (Charles River Laboratories, France) were used. The mating day was considered embryonic day 0. Pregnant females at the development stage of interest were killed by cervical dislocation, following the European Community Council directive and the National Institutes of Health guidelines for the care and use of laboratory animals. Experiments were also approved by the local ethics committees.

### Neuronal primary culture

E16 mouse brains were dissected out to obtain hippocampal neuronal cultures in PBS containing 0.6% glucose. The brains were then dissociated by incubation for 12 min at 37°C with 0.05% trypsin (Invitrogen) and treated for 10 min with DNase (Roche Diagnostics); tissue pieces were dissociated by gentle sweeping. Cells were then counted and seeded at a density of 50,000 neurons per dish in 4-well plates on coverslips pre-coated with poly-D lysine (Sigma) for immunocytochemistry and at a density of 3 million neurons per well in 6-well plates and 10^7^ cells in 90-mm plates for biochemical analysis. Cells were cultured for 3 days in Neurobasal medium containing 1% glutamax and 2% B27 supplement. Primary cultures were incubated with BDNF for 0, 15, and 30 min (50 ng/ml, Promega).

For Nodose primary cultures, E14 mouse embryos were dissected in F12 media (Gibco-BRL). Ganglia were dissociated for 10 min at 37°C with 0.05% trypsin (Invitrogen), and the tissue pieces were dissociated by gentle sweeping. Cells were then counted and seeded at a density of 5,000 neurons per dish in 4-well plates on coverslips pre-coated with polyornithine (Sigma, 0.5% overnight) and laminin (20 ug/ml for 2 h) for immunocytochemistry. The neurons were incubated for 24 h in a defined media containing Dulbecco’s Modified Eagle’s Medium (DMEM, Gibco-BRL), 10% Fetal Bovine Serum (FBS), 0.54% glucose, 1% glutamine, 1% Penicillin/ streptomycin, N2 and B27, and supplemented with 50 ng/ml BDNF (Promega). The day after, primary cultures were deprived for 2 h and then treated with 100 ng BDNF or control medium for 0, 5, 15 and 30 min.

### Explant cultures

Hippocampal explants were obtained from E15 brains, sectioned using a tissue chopper. Small tissue explants (300–400 µm thick) were obtained from the CA1/CA3 regions [77]. Hippocampal explants were cultured in Neurobasal medium (Gibco-BRL) plus 0.54% glucose, 0.032% NaH2CO3, L-glutamine, and 10% Horse Serum and supplemented with B27. Nodose and DRG explants (E14), obtained as above, were embedded in collagen gels for 1 day or cultured on polyornithine-laminin dishes in DMEM (Gibco-BRL) plus medium supplemented with 0.54% glucose, 0.032% NaH2CO3, L-glutamine, B27, N2 and BDNF (50 ng/ml, Promega), and NT3 (50 ng/ml, PreproTech) or NGF (50 ng/ml, Sigma). Explant cultures were treated with 25 nM BoNT/A (Metabiologics, INC), 15 nM BoNT/C1 (Metabiologics, INC) or 2 nM TeNT (Sigma) for 24 h.

### Bodipy labeling

Nodose explants were incubated on day 2 and day 3 respectively, with the Bodipy-labeled-ceramide–BSA complex (Invitrogen). To allow internalization and accumulation of the labeled lipid complex in the Golgi apparatus, explants were incubated for 30 min at 37°C in their own medium and washed 5 times. To allow transport to the cell surface, they were further incubated at 37°C for 2 or 3 h [51, 78]. The nodose explants were then time-course treated with BDNF for 0, 15 and 30 min. After the treatment, they were fixed with 4% paraformaldehyde and 0.5% glutaraldehyde/PBS 0.1M for 30 min and washed with PBS 0.1M several times before the coverslips were mounted with Mowiol. The binding of Bodipy to cells was monitored with single-line excitation at 543 nm and 488 nm using a TCS SP2 confocal microscope.

### Bodipy quantification

Quantitative analysis of the confocal images of growth cones was performed by measuring the average pixel intensity of each image with an ImageJ macro. The macro transformed all the images (red and green) into a 32-bit depth and then ran a threshold between 10 to 255 gray levels to avoid background from 0 to 10 gray levels. Finally, we measured the area and the mean gray value for each channel. The average pixel intensity was then calculated from this value on the basis of the ratio between red and green channels. The values were classified in function of the treatment time. At least 5 separate experiments (usually 7–8) were carried out for each condition. The number of axonal growth cones counted is shown in the figure legends.

### HEK 293T culture and transfection

HEK293 cells were cultured in DMEM, 10% FBS, 0.5% glutamine and 1% penicillin/ streptomycin. All cells were maintained in 5% CO2 at 37°C. For transfection, cells were cultured overnight to 60–70% confluence in 90-mm culture plates. The transfection was performed in Opti-MEM medium with Lipofectamine Reagent Plus, following the manufacturer’s instructions (Invitrogen, Carlsbad, CA). Cells were transfected with different combinations of cDNAs: pEF-BOS-SNAP25-FLAG and pEF-BOS-VAMP2-FLAG (kindly provided by Dr. M. Fukuda, RIKEN, Japan); TI-VAMP (TrueClone, Origene); pcDNA-TrkA-HA (kindly provided by Dr. D. Martin-Zanca); pEGFPc1-TrkB (kindly provided by Dr. RA Segal, Harvard Medical School, USA); and pcDNA-TrkC-myc (kindly provided by Dr. RA Barker, Montreal Neurological Institute, Canada); pCMVtag3a-TM-JX-TK-Y817-TrkB (TrkB_TM-JX-TK-Y817_); pCMVtag3a-TM-JX-TK-TrkB (TrkB_TM-JX-TK_); pCMVtag3a-JX-TK-TrkB (TrkB_JX-TK-Y817_); and pCMVtag3a-TK-TrkB (TrkB_TK_).

The TrkB truncated fragments were obtained by PCR amplification of pEGFPc1-TrkB using specific primers and subsequently cloned into EcoRI/HindIII sites in the pCMVtag3a expression vector (Clontech). The TrkB_TM-JX-TK-Y817_ fragment was obtained by PCR amplification of pEGFPc1-TrkB using the following primers: Forward 5′TATATGAATTCTATGCATCTCTCGGTCTATGCT3′ and Backward 5′ATATATAAGCTTCTAGCCTAGGATGTCCAGGTA3′). The TrkB_TM-JX-TK_ fragment was obtained by PCR amplification of pEGFPc1-TrkB using the following primers: Forward 5′TATATGAATTCTATGCATCTCTCGGTCTATGCT3′ and Backward 5′ATATATAAGCTTCTACGCCTTCGCCAAGTTCTG3′. The TrkB_JX-TK-y817_ fragment was obtained by PCR amplification of pEGFPc1-TrkB using the following primers: Forward 5′TATATGAATTCTATGAAGTTGGCGAGACATTCC3′ and Backward 5′ATATATAAGCTTCTAGCCTAGGATGTCCAGGTA3′. Finally, the TrkB_TK_ fragment was obtained by PCR amplification of pEGFPc1-TrkB using the following primers: Forward 5′TATATGAATTCTATGCACAACATCGTTCTGAAG3′ and Backward 5′ATATATAAGCTTCTACGCCTTCGCCAAGTTCTG3′.

Full-length Sytx1A or truncated DNAs (Sytx1ACYT, Sytx1AHabc, Sytx1AH3TM, Sytx1AH3, and Sytx1ATM) were cloned into EcoRI sites in pEGFPC1 fusion mammalian protein expression vector (Clontech), as described [18].

### Primary culture and explant transfection

Using the NeonTM transfection system (Invitrogen), nodose-dissociated primary cultures were transfected with one of the following DNAs: pEGFPC1, Sytx1AH3TMEGFP, shRNA TI-VAMP D06 (5′CCGGGCACTTCCTTATGCTATGAATCTCGAGATTCATAGCATAAGGAAGTGCTTTTTG3′, MISSION iRNA, Sigma) (TI-VAMP shRNAD06) and shRNA TI-VAMP D07 (5′CCGGCTTACTCACATGGCAATTATTCTCGAGAATAATTGCCATGTGAGTAAGTTTTTG3′, MISSION iRNA, Sigma) (TI-VAMP shRNAD07). For Sytx1 RNAi experiments, we used a pSUPER.retro.puro plasmid (OligoEngine, Seattle, WA, USA) and specific oligonucleotides of the Sytx1A sequence: 5′GATCCCCCCAAGAAGGCCGTCAAGTATTCAAGAGATACTTGACGGCCTTCTTGGTTTTT3′ (Forward), and 5′AGCTAAAAACCAAGAAGGCCGTCAAGTATCTCTTGAATACTTGACGGCCTTCTTGGGGG3′ (Reverse) [17] (Sytx1AshRNA1) and a shRNA Sytx-1 (5′CCGGGAAAGCCATCGAGCAAGGAATCTCGAGATTCCTTGCTCGATGGCTTTCTTTTTG3′), MISSION iRNA, Sigma) (Sytx1AshRNA2).

The cells and explants were washed twice with PBS 0.1M and then electroporated with two pulses of 1200V for 20 sec, followed by three pulses of 500V for 30 sec. The explants were then plated in collagen, while the primary culture was plated in polyornithine-laminin pre-coated coverslips at a cell density of 5,000, both in DMEM plus medium supplemented with 0.54% glucose, 0.032% NaH2CO3, L-glutamine, B27, N2 and BDNF (50 ng/ml, Promega).

### Immunoprecipitation assays and immunoblots

Embryonic and adult brains, as well as transiently transfected HEK293 cells and primary cultures, were collected in hypotonic lysis buffer (10 mM KCl, 15 mM MgCl2, 10 mM Tris pH 7.2, 5 mM Na4H2PO4, 1% Triton X-100, 10 µg/ml Leupeptin, 10 µg/ml Aprotinin, 1 mM PMSF) containing a protease inhibitor cocktail (Roche). Thereafter, cells were centrifuged at 13,000 rpm for 20 min, and the pellet was discarded. The supernatant was analyzed by Western blot or subjected to immunoprecipitation. For tissue extracts, samples were weighed and 3 volumes of lysis buffer was added. The mixture was processed using a Polytron homogenizer, and insoluble material was removed by centrifugation at 13,000rpm for 20 min.

Protein concentration was determined by means of the Bradford assay. For the immunoprecipitation assays, 200–400 µg of total protein per sample was used. Brain lysate homogenates were incubated overnight at 4°C with anti-TrkB (1:500; Upstate), anti-Sytx1 (HPC-1 clone 1:500-1000; Sigma), anti-SNAP25 (SMI-81 clone 1:500; Sternberger Meyer), or anti-VAMP2 (1:500; Synaptic Systems) in IP buffer (10 mM Tris pH 7, 140 mM NaCl, 1.5 mM EDTA). Transfected HEK293 cell homogenates were incubated with anti-FLAG (1:1000; Sigma), anti-myc (1:1000; Roche), anti-HA (1:500, Roche), anti-GFP (1:1000; Molecular Probes), or anti-TI-VAMP (1:200 Abcam) antibodies. All the assays were performed using Protein G-sepharose beads (Sigma) for 2 h at 4°C. After three washes, 1 volume of 3x Lamelli loading buffer (75 mM pH 6.5, 0.5 mM β-mercaptoethanol, 0.5% SDS, 10% glycerol, and 0.000125% bromophenol blue)/SDS-sample buffer was added to the beads, and the proteins were analyzed by SDS-PAGE and Western blot. Samples were run in polyacrylamide gel at the opportune concentration at 120V. Afterwards, proteins were transferred onto nitrocellulose membranes overnight at 135 mA at 4°C. The membranes were blocked with 5% non-fat dry milk in Tris-HCl-buffered saline (TBS) containing 0.1% Tween 20, and incubated overnight at 4°C with anti-TrkB (1:1000, Upstate), anti-Sytx1 (HPC-1 clone 1:500-1000, Sigma), anti-SNAP25 (SMI-81 clone 1:500 Sternberger Meyer), anti-VAMP2 (1:500, Synaptic Systems), mouse anti-TI-VAMP (1:200 Abcam), rabbit anti-GFP (1:2000, Invitrogen), mouse anti-FLAG (1:1000, Sigma), mouse anti-HA (1:1000, Roche), or mouse anti-myc (1:1000, Roche) antibodies. After incubation with secondary antibodies, blots were developed following the ECL method (Amersham Pharmacia Biotech).

To normalize the data, we represented the ratios between the amount of co-immunoprecipitated protein (e.g., TrkB) and the immunoprecipitating protein (Sytx1) (expressed as percentage association with respect to controls).

### Immunocytochemistry

Nodose explants, DRG explants, and primary cultures were fixed with paraformaldehyde 4% PBS 0.1M (pH 7.4) for 30 min and washed with PBS 0.1M several times. The cultures were then permeabilized and blocked for 3 h with PBS, Normal Goat Serum 2% and Triton X-100 0.1% (Sigma). Finally, they were incubated overnight at 4°C with primary antibody against neuron-specific class βIIITubulin (clone TUJ-1, 1:4000; Covance), rabbit anti-GFP (1:500, Invitrogen), mouse anti-Sytx1 (1:500), rabbit anti-TrkB (1:500, Upstate), or TI-VAMP (1:250, Abcam), followed by incubation with Alexa-fluor 488- and Alexa-fluor 568-coupled corresponding secondary antibodies (1:500) and DAPI (Sigma) for 2 h at room temperature. The coverslips were then washed and mounted on glass slides with Mowiol.

### Quantification of explants

The images were captured by an E1000 fluorescence microscope from Nikon, using a 4X0.13NA and 10X 0.30 NA lens. Metamorph software and a CoolSnap fix scientific CCD camera were used for acquisition. Explants treated with toxins were quantified using Image J by calculating the area in pixels occupied by the axons. A background pixel level was defined and used to subtract the former value, and upper and lower density limits were defined for each explant. Once the segmentation range was defined, any pixels falling within this range were automatically counted. To quantify the area occupied by neurites emanating from explants, the tissue explant was manually drawn and excluded from the picture, thereby giving an area measurement for the pixels occupied by the neurites alone. The values obtained were plotted on a bar chart with their standard deviation. Data were collected from at least three independent experiments (3–7 experiments per condition). The number of cultures quantified is shown in the Figures.

### Quantification of immunocytochemistry experiments

Confocal images of axonal growth cones (defined as the terminal portion of the axons, having a triangular shape and being notably thicker than the shaft of the axon) were taken with a Leica TCS SPE (Leica Microsystems GmbH, Manheim, Germany) confocal scanning microscope adapted to an upright LEICA DM 2500 microscope. Samples were scanned using 40x 1.15 NA and 63x 1.3NA oil objectives. We used a zoom ranging from 1 to 3.5 to analyze intracellular regions and the LAS AF software from Leica.

Fluorescent images were processed using open software Image J. PDM images ((red intensity-mean red intensity)×(green intensity-mean green intensity)) were calculated and normalized for each pixel of an image and expressed as the degree of co-localization of two signals, with a value varying from –1 to +1, where –1 indicates maximal exclusion, negative values asynchronous localization, positive values synchronous localization (or co-localization), and +1 maximal co-localization. PDM images and Mander’s overlap coefficients were calculated using ImageJ “Intensity Correlation Analysis plug-in” [79].

### Quantification of Nodose primary cultures

The entire images of coverslips were detected by an automated inverted fluorescence wide-field microscope with high content screening ScanR from Olympus IX81 using a 20x objective lens and a Hamamatsu Orca-ER digital camera (12bit acquisition, 1344*1024 pixels chip, and 6.45 μm/pixel). All the captured images were used to run an ImageJ plug-in “Stitch grid of images” and obtain a single mosaic image for each coverslip. The total image of the coverslips was used to calculate the total length of each axon, while the Neuron J plug-in was used to calculate ramification. The total length of each experimental group was normalized to control conditions.

### Statistics

Data were expressed as means ± SEM and were analyzed using the Kruskal-Wallis test, followed by the Bonferroni Post-hoc test (Statgraphics Centurion XVII.I).

### Data availability

The datasets generated or analyzed during the current study are available from the corresponding authors on request.

## SUPPLEMENTARY MATERIALS FIGURES



## Abbreviations

BDNF: brain-derived neurotrophic factor
BoNT/A: Botulinum Toxin A
BoNT/C1: Botulinum Toxin C1
DRG: dorsal root ganglion
NGF: Nerve growth factor
Nrp1: Neuropilin 1
NT-3: neurotrophin-3
NT-4: neurotrophin-4
PlexA1: Plexin A1
PTB: phosphotyrosine binding
Sema3A: Semaphorin 3A
SH2: Src-homology-2
SNAP25: 25-kDa soluble N-ethylmaleimide-sensitive factor attachment protein
Sytx1: Syntaxin 1
TeNT: tetanus toxin
TI-VAMP: Tetanus Neurotoxin Insensitive Vesicle-Associated Membrane Protein
Trk: tropomyosin-related kinase
VAMP2: Vesicle-associated membrane protein 2

## References

[1] Alberts P, Rudge R, Irinopoulou T, Danglot L, Gauthier-Rouvière C, Galli T (2006). Cdc42 and actin control polarized expression of TI-VAMP vesicles to neuronal growth cones and their fusion with the plasma membrane. Mol Biol Cell.

[2] Igarashi M, Tagaya M, Komiya Y (1997). The soluble N-ethylmaleimide-sensitive factor attached protein receptor complex in growth cones: molecular aspects of the axon terminal development. J Neurosci.

[3] Martinez-Arca S, Coco S, Mainguy G, Schenk U, Alberts P, Bouillé P, Mezzina M, Prochiantz A, Matteoli M, Louvard D, Galli T (2001). A common exocytotic mechanism mediates axonal and dendritic outgrowth. J Neurosci.

[4] Pfenninger KH, Laurino L, Peretti D, Wang X, Rosso S, Morfini G, Cáceres A, Quiroga S (2003). Regulation of membrane expansion at the nerve growth cone. J Cell Sci.

[5] Tojima T, Akiyama H, Itofusa R, Li Y, Katayama H, Miyawaki A, Kamiguchi H (2007). Attractive axon guidance involves asymmetric membrane transport and exocytosis in the growth cone. Nat Neurosci.

[6] Sabo SL, McAllister AK (2003). Mobility and cycling of synaptic protein-containing vesicles in axonal growth cone filopodia. Nat Neurosci.

[7] Steiner P, Sarria JC, Huni B, Marsault R, Catsicas S, Hirling H (2002). Overexpression of neuronal Sec1 enhances axonal branching in hippocampal neurons. Neuroscience.

[8] Chen YA, Scheller RH (2001). SNARE-mediated membrane fusion. Nat Rev Mol Cell Biol.

[9] Martinez-Arca S, Alberts P, Zahraoui A, Louvard D, Galli T (2000). Role of tetanus neurotoxin insensitive vesicle-associated membrane protein (TI-VAMP) in vesicular transport mediating neurite outgrowth. J Cell Biol.

[10] Lowery LA, Van Vactor D (2009). The trip of the tip: understanding the growth cone machinery. Nat Rev Mol Cell Biol.

[11] Gomez TM, Letourneau PC (2014). Actin dynamics in growth cone motility and navigation. J Neurochem.

[12] Vitriol EA, Zheng JQ (2012). Growth cone travel in space and time: the cellular ensemble of cytoskeleton, adhesion, and membrane. Neuron.

[13] Tojima T, Kamiguchi H (2015). Exocytic and endocytic membrane trafficking in axon development. Dev Growth Differ.

[14] Filippini F, Rossi V, Galli T, Budillon A, D’Urso M, D’Esposito M (2001). Longins: a new evolutionary conserved VAMP family sharing a novel SNARE domain. Trends Biochem Sci.

[15] Rossi V, Picco R, Vacca M, D’Esposito M, D’Urso M, Galli T, Filippini F (2004). VAMP subfamilies identified by specific R-SNARE motifs. Biol Cell.

[16] Alberts P, Rudge R, Hinners I, Muzerelle A, Martinez-Arca S, Irinopoulou T, Marthiens V, Tooze S, Rathjen F, Gaspar P, Galli T (2003). Cross talk between tetanus neurotoxin-insensitive vesicle-associated membrane protein-mediated transport and L1-mediated adhesion. Mol Biol Cell.

[17] Cotrufo T, Andrés RM, Ros O, Pérez-Brangulí F, Muhaisen A, Fuschini G, Martínez R, Pascual M, Comella JX, Soriano E (2012). Syntaxin 1 is required for DCC/Netrin-1-dependent chemoattraction of migrating neurons from the lower rhombic lip. Eur J Neurosci.

[18] Cotrufo T, Pérez-Brangulí F, Muhaisen A, Ros O, Andrés R, Baeriswyl T, Fuschini G, Tarrago T, Pascual M, Ureña J, Blasi J, Giralt E, Stoeckli ET, Soriano E (2011). A signaling mechanism coupling netrin-1/deleted in colorectal cancer chemoattraction to SNARE-mediated exocytosis in axonal growth cones. J Neurosci.

[19] Danglot L, Zylbersztejn K, Petkovic M, Gauberti M, Meziane H, Combe R, Champy MF, Birling MC, Pavlovic G, Bizot JC, Trovero F, Della Ragione F, Proux-Gillardeaux V (2012). Absence of TI-VAMP/Vamp7 leads to increased anxiety in mice. J Neurosci.

[20] Tojima T, Hines JH, Henley JR, Kamiguchi H (2011). Second messengers and membrane trafficking direct and organize growth cone steering. Nat Rev Neurosci.

[21] Tojima T, Itofusa R, Kamiguchi H (2010). Asymmetric clathrin-mediated endocytosis drives repulsive growth cone guidance. Neuron.

[22] Tojima T, Itofusa R, Kamiguchi H (2014). Steering neuronal growth cones by shifting the imbalance between exocytosis and endocytosis. J Neurosci.

[23] Bibel M, Barde YA (2000). Neurotrophins: key regulators of cell fate and cell shape in the vertebrate nervous system. Genes Dev.

[24] Bothwell M (1995). Functional interactions of neurotrophins and neurotrophin receptors. Annu Rev Neurosci.

[25] Reichardt LF (2006). Neurotrophin-regulated signalling pathways. Philos Trans R Soc Lond B Biol Sci.

[26] Skaper SD (2012). The neurotrophin family of neurotrophic factors: an overview. Methods Mol Biol.

[27] Arévalo JC, Wu SH (2006). Neurotrophin signaling: many exciting surprises!. Cell Mol Life Sci.

[28] Huang EJ, Reichardt LF (2001). Neurotrophins: roles in neuronal development and function. Annu Rev Neurosci.

[29] Park H, Poo MM (2013). Neurotrophin regulation of neural circuit development and function. Nat Rev Neurosci.

[30] Leal G, Afonso PM, Salazar IL, Duarte CB (2015). Regulation of hippocampal synaptic plasticity by BDNF. Brain Res.

[31] Markus A, Patel TD, Snider WD (2002). Neurotrophic factors and axonal growth. Curr Opin Neurobiol.

[32] Tucker KL, Meyer M, Barde YA (2001). Neurotrophins are required for nerve growth during development. Nat Neurosci.

[33] Colombo F, Racchetti G, Meldolesi J (2014). Neurite outgrowth induced by NGF or L1CAM via activation of the TrkA receptor is sustained also by the exocytosis of enlargeosomes. Proc Natl Acad Sci U S A.

[34] Martínez A, Alcántara S, Borrell V, Del Río JA, Blasi J, Otal R, Campos N, Boronat A, Barbacid M, Silos-Santiago I, Soriano E (1998). TrkB and TrkC signaling are required for maturation and synaptogenesis of hippocampal connections. J Neurosci.

[35] Thiele CJ, Li Z, McKee AE (2009). On Trk—the TrkB signal transduction pathway is an increasingly important target in cancer biology. Clin Cancer Res.

[36] Vaishnavi A, Le AT, Doebele RC (2015). TRKing down an old oncogene in a new era of targeted therapy. Cancer Discov.

[37] Barrecheguren PJ, Ros O, Cotrufo T, Kunz B, Soriano E, Ulloa F, Stoeckli ET, Araújo SJ (2017). SNARE proteins play a role in motor axon guidance in vertebrates and invertebrates. Dev Neurobiol.

[38] Fernández-Nogueira P, Bragado P, Almendro V, Ametller E, Rios J, Choudhury S, Mancino M, Gascón P (2016). Differential expression of neurogenes among breast cancer subtypes identifies high risk patients. Oncotarget.

[39] Ulloa F, Gonzàlez-Juncà A, Meffre D, Barrecheguren PJ, Martínez-Mármol R, Pazos I, Olivé N, Cotrufo T, Seoane J, Soriano E (2015). Blockade of the SNARE protein syntaxin 1 inhibits glioblastoma tumor growth. PLoS One.

[40] Huber AB, Kolodkin AL, Ginty DD, Cloutier JF (2003). Signaling at the growth cone: ligand-receptor complexes and the control of axon growth and guidance. Annu Rev Neurosci.

[41] Kerstein PC, Nichol RH, Gomez TM (2015). Mechanochemical regulation of growth cone motility. Front Cell Neurosci.

[42] Kofuji T, Fujiwara T, Sanada M, Mishima T, Akagawa K (2014). HPC-1/syntaxin 1A and syntaxin 1B play distinct roles in neuronal survival. J Neurochem.

[43] Varoqueaux F, Sigler A, Rhee JS, Brose N, Enk C, Reim K, Rosenmund C (2002). Total arrest of spontaneous and evoked synaptic transmission but normal synaptogenesis in the absence of Munc13-mediated vesicle priming. Proc Natl Acad Sci U S A.

[44] Verhage M, Maia AS, Plomp JJ, Brussaard AB, Heeroma JH, Vermeer H, Toonen RF, Hammer RE, van den Berg TK, Missler M, Geuze HJ, Südhof TC (2000). Synaptic assembly of the brain in the absence of neurotransmitter secretion. Science.

[45] Zimmermann J, Trimbuch T, Rosenmund C (2014). Synaptobrevin 1 mediates vesicle priming and evoked release in a subpopulation of hippocampal neurons. J Neurophysiol.

[46] Fariñas I, Jones KR, Backus C, Wang XY, Reichardt LF (1994). Severe sensory and sympathetic deficits in mice lacking neurotrophin-3. Nature.

[47] Liebl DJ, Tessarollo L, Palko ME, Parada LF (1997). Absence of sensory neurons before target innervation in brain-derived neurotrophic factor-, neurotrophin 3-, and TrkC-deficient embryonic mice. J Neurosci.

[48] Schiavo G, Matteoli M, Montecucco C (2000). Neurotoxins affecting neuroexocytosis. Physiol Rev.

[49] Blasi J, Chapman ER, Link E, Binz T, Yamasaki S, De Camilli P, Südhof TC, Niemann H, Jahn R (1993). Botulinum neurotoxin A selectively cleaves the synaptic protein SNAP-25. Nature.

[50] Blasi J, Chapman ER, Yamasaki S, Binz T, Niemann H, Jahn R (1993). Botulinum neurotoxin C1 blocks neurotransmitter release by means of cleaving HPC-1/syntaxin. EMBO J.

[51] Pagano RE, Martin OC, Kang HC, Haugland RP (1991). A novel fluorescent ceramide analogue for studying membrane traffic in animal cells: accumulation at the Golgi apparatus results in altered spectral properties of the sphingolipid precursor. J Cell Biol.

[52] Huber LA, Dupree P, Dotti CG (1995). A deficiency of the small GTPase rab8 inhibits membrane traffic in developing neurons. Mol Cell Biol.

[53] Gallo G, Letourneau P (2002). Axon guidance: proteins turnover in turning growth cones. Curr Biol.

[54] Gallo G, Letourneau PC (2004). Regulation of growth cone actin filaments by guidance cues. J Neurobiol.

[55] Long KE, Lemmon V (2000). Dynamic regulation of cell adhesion molecules during axon outgrowth. J Neurobiol.

[56] Suter DM, Schaefer AW, Forscher P (2004). Microtubule dynamics are necessary for SRC family kinase-dependent growth cone steering. Curr Biol.

[57] Zheng JQ, Wan JJ, Poo MM (1996). Essential role of filopodia in chemotropic turning of nerve growth cone induced by a glutamate gradient. J Neurosci.

[58] Bentley D, O’Connor TP (1994). Cytoskeletal events in growth cone steering. Curr Opin Neurobiol.

[59] Deppmann CD, Mihalas S, Sharma N, Lonze BE, Niebur E, Ginty DD (2008). A model for neuronal competition during development. Science.

[60] Rodgers EE, Theibert AB (2002). Functions of PI 3-kinase in development of the nervous system. Int J Dev Neurosci.

[61] Laurino L, Wang XX, de la Houssaye BA, Sosa L, Dupraz S, Cáceres A, Pfenninger KH, Quiroga S (2005). PI3K activation by IGF-1 is essential for the regulation of membrane expansion at the nerve growth cone. J Cell Sci.

[62] Chen MJ, Nguyen TV, Pike CJ, Russo-Neustadt AA (2007). Norepinephrine induces BDNF and activates the PI-3K and MAPK cascades in embryonic hippocampal neurons. Cell Signal.

[63] Schratt G, Philippar U, Hockemeyer D, Schwarz H, Alberti S, Nordheim A (2004). SRF regulates Bcl-2 expression and promotes cell survival during murine embryonic development. EMBO J.

[64] Cox LJ, Hengst U, Gurskaya NG, Lukyanov KA, Jaffrey SR (2008). Intra-axonal translation and retrograde trafficking of CREB promotes neuronal survival. Nat Cell Biol.

[65] Lin AC, Holt CE (2008). Outsourcing CREB translation to axons to survive. Nat Cell Biol.

[66] Craig AM, Wyborski RJ, Banker G (1995). Preferential addition of newly synthesized membrane protein at axonal growth cones. Nature.

[67] Pfenninger KH (2009). Plasma membrane expansion: a neuron’s Herculean task. Nat Rev Neurosci.

[68] Winckler B, Mellman I (2010). Trafficking guidance receptors. Cold Spring Harb Perspect Biol.

[69] Zylbersztejn K, Galli T (2012). [Membrane traffic, a new actor in axon guidance]. [Article in French]. Med Sci (Paris).

[70] Ros O, Cotrufo T, Martínez-Mármol R, Soriano E (2015). Regulation of patterned dynamics of local exocytosis in growth cones by netrin-1. J Neurosci.

[71] Washbourne P, Thompson PM, Carta M, Costa ET, Mathews JR, Lopez-Benditó G, Molnár Z, Becher MW, Valenzuela CF, Partridge LD, Wilson MC (2002). Genetic ablation of the t-SNARE SNAP-25 distinguishes mechanisms of neuroexocytosis. Nat Neurosci.

[72] Vilinsky I, Stewart BA, Drummond J, Robinson I, Deitcher DL (2002). A Drosophila SNAP-25 null mutant reveals context-dependent redundancy with SNAP-24 in neurotransmission. Genetics.

[73] Osen-Sand A, Staple JK, Naldi E, Schiavo G, Rossetto O, Petitpierre S, Malgaroli A, Montecucco C, Catsicas S (1996). Common and distinct fusion proteins in axonal growth and transmitter release. J Comp Neurol.

[74] Schoch S, Deák F, Königstorfer A, Mozhayeva M, Sara Y, Südhof TC, Kavalali ET (2001). SNARE function analyzed in synaptobrevin/VAMP knockout mice. Science.

[75] Vardar G, Chang S, Arancillo M, Wu YJ, Trimbuch T, Rosenmund C (2016). Distinct Functions of Syntaxin-1 in Neuronal Maintenance, Synaptic Vesicle Docking, and Fusion in Mouse Neurons. J Neurosci.

[76] Ros O, Barrecheguren PJ, Cotrufo T, Schaettin M, Roselló-Busquets C, Vílchez-Acosta A, Hernaiz-Llorens M, Martínez-Marmol R, Ulloa F, Stoeckli ET, Araújo SJ, Soriano E (2018). A conserved role for Syntaxin-1 in pre- and post-commissural midline axonal guidance in fly, chick, and mouse. PLoS Genet.

[77] Barallobre MJ, Del Río JA, Alcántara S, Borrell V, Aguado F, Ruiz M, Carmona MA, Martín M, Fabre M, Yuste R, Tessier-Lavigne M, Soriano E (2000). Aberrant development of hippocampal circuits and altered neural activity in netrin 1-deficient mice. Development.

[78] Paglini G, Peris L, Diez-Guerra J, Quiroga S, Cáceres A (2001). The Cdk5-p35 kinase associates with the Golgi apparatus and regulates membrane traffic. EMBO Rep.

[79] Li Q, Lau A, Morris TJ, Guo L, Fordyce CB, Stanley EF (2004). A syntaxin 1, Galpha(o), and N-type calcium channel complex at a presynaptic nerve terminal: analysis by quantitative immunocolocalization. J Neurosci.

