# Supplementary material for: Syntaxin-1/TI-VAMP SNAREs interact with Trk receptors and are required for neurotrophin-dependent outgrowth
Source: Oncotarget. 2018 Nov 13;9(89):35922–40. doi: 10.18632/oncotarget.26307 (PMC6267591; doi:10.18632/oncotarget.26307)
Supplement: Supplementary file 1 [file oncotarget-09-35922-s001.pdf]

Syntaxin-1/TI-VAMP SNAREs interact with Trk receptors and are required for neurotrophin-dependent outgrowth

SUPPLEMENTARY MATERIALS

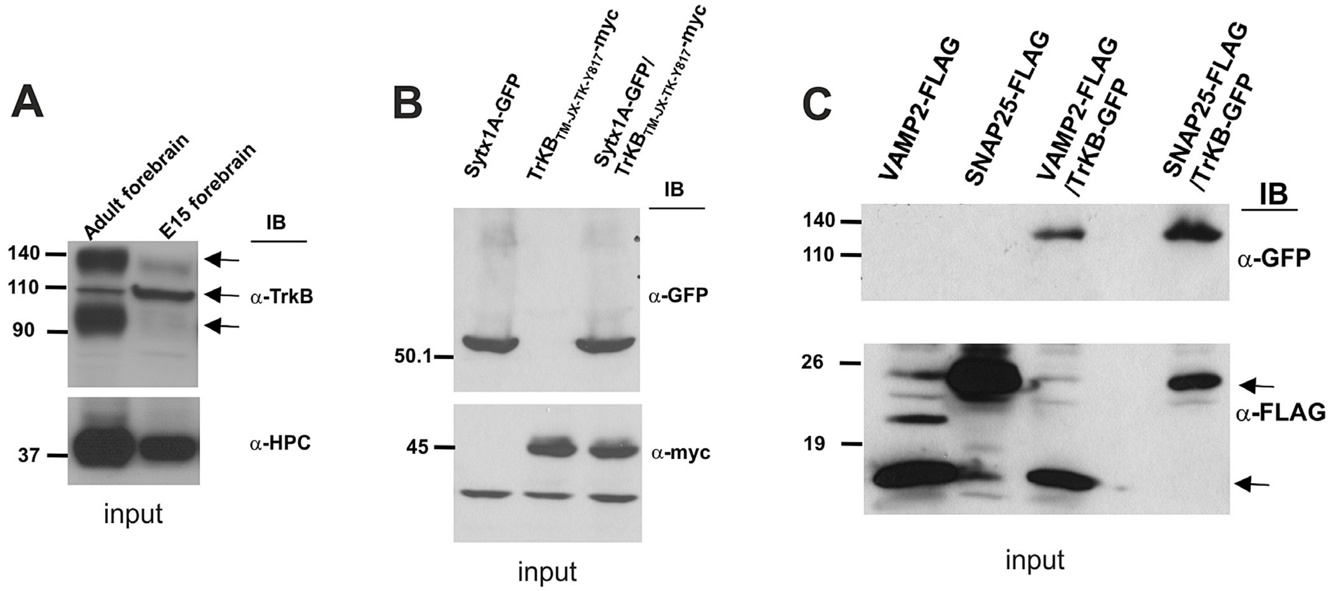

Supplementary Figure 1: Examples of inputs of TrkB and Sytx1 immunoprecipitation experiments, corresponding to the experiments shown in Figures 1A, 1B, 2A and 2B. Experiments are from E15 and adult forebrain homogenates (20 µg of total protein) (**A**) and from HEK293 cells transfected with pSytx1AEGFP alone or together with pCMVtag3A-$TrkB_{TM-JX-TK-Y817}$ (**B**) or transfected with pEF-BOS-SNAP25-FLAG alone or together with pEGFPC1-TrkB or with pEF-BOS-VAMP2-FLAG alone or together with pEGFPC1-TrkB (**C**).

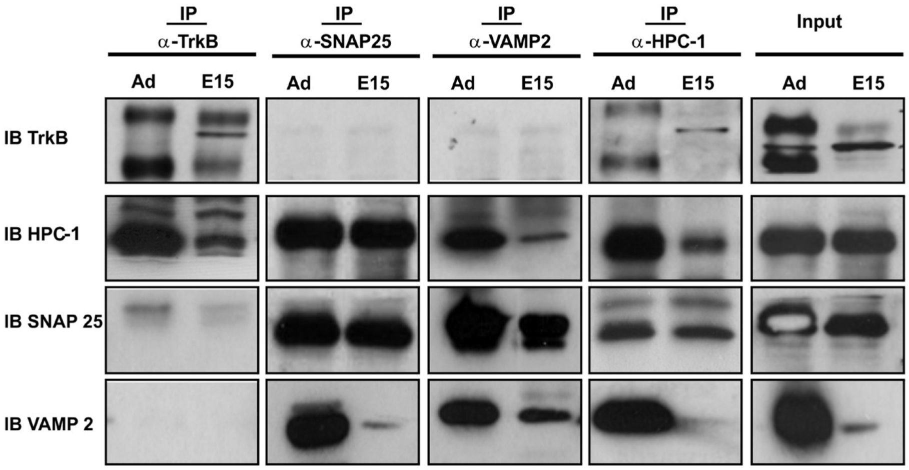

Supplementary Figure 2: TrkB, SNAP25 and VAMP2 immunoprecipitation of E15 forebrain and adult homogenates (100 µg of total protein). To allow comparison, we have also included panels of Figure 1A for Sytx-1-TrkB co-immunoprecipitation. Last row: inputs using the antibody of each protein. No co-immunoprecipitation was detected between TrkB and SNAP25 or VAMP2.

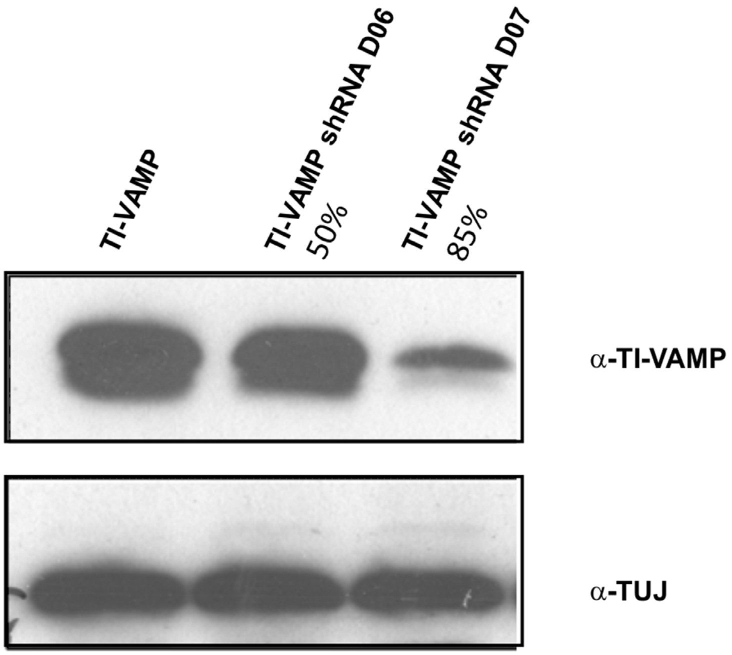

Supplementary Figure 3: Downregulation of TI-VAMP. Expression of TI-VAMP alone and together with the TI-VAMP shRNA D06 or TI-VAMP shRNA D07 in HEK293 cells, showing that these shRNAs show a 50% and 85% decrease the expression of TI-VAMP, respectively.
